# Stochastic sequestration dynamics: a minimal model with extrinsic noise for bimodal distributions and competitors correlation

**DOI:** 10.1038/s41598-018-28647-9

**Published:** 2018-07-10

**Authors:** Marco Del Giudice, Carla Bosia, Silvia Grigolon, Stefano Bo

**Affiliations:** 1 Department of Applied Science and Technology, Politecnico di Torino corso Duca degli Abruzzi 24, Turin, IT-10129 Italy; 2 Italian Institute for Genomic Medicine, via Nizza 52, I-10126 Torino, Italy; 3 The Francis Crick Institute, 1, Midland Road, London, NW1 1AT United Kingdom; 4 Nordita, Royal Institute of Technology and Stockholm University, Roslagstullsbacken 23, SE-106 91 Stockholm, Sweden

## Abstract

Many biological processes are known to be based on molecular sequestration. This kind of dynamics involves two types of molecular species, namely targets and sequestrants, that bind to form a complex. In the simple framework of mass-action law, key features of these systems appear to be threshold-like profiles of the amounts of free molecules as a function of the parameters determining their possible maximum abundance. However, biochemical processes are probabilistic and take place in stochastically fluctuating environments. How these different sources of noise affect the final outcome of the network is not completely characterised yet. In this paper we specifically investigate the effects induced by a source of extrinsic noise onto a minimal stochastic model of molecular sequestration. We analytically show how bimodal distributions of the targets can appear and characterise them as a result of noise filtering mediated by the threshold response. We then address the correlations between target species induced by the sequestrant and discuss how extrinsic noise can turn the negative correlation caused by competition into a positive one. Finally, we consider the more complex scenario of competitive inhibition for enzymatic kinetics and discuss the relevance of our findings with respect to applications.

## Introduction

**Figure 1:**
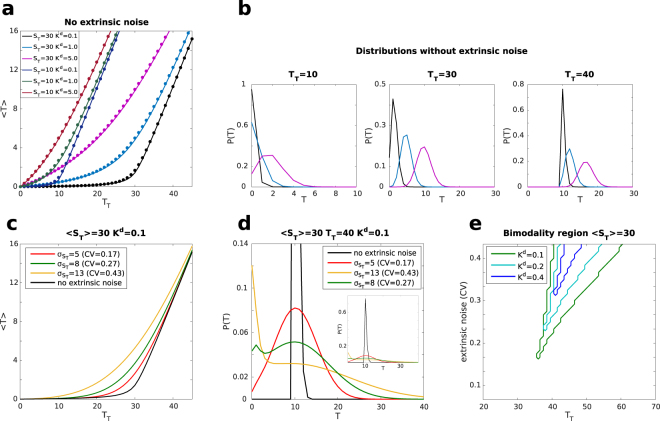
(**a**) 〈*T*〉 vs *T*_*T*_ for *S*_*T*_ = 10 and *S*_*T*_ = 30. Solid lines are the solution of the master eq. (12) while symbols the one of the rate eq. (6). *K*^*d*^ takes the values 0.1, 1.0, 5.0 (curves with lower *K*^*d*^ are below the ones with a higher one). (**b**) Examples of probability distributions from the solution of the master eq. (10). *S*_*T*_ = 30, *K*^*d*^ assumes the values: 5.0 (purple), 1.0 (cyan) and 0.1 (black). From left to right *T*_*T*_ = 10, 30, 40. (**c**) 〈*T*〉 vs *T*_*T*_ in presence of extrinsic noise on *S*_*T*_ as obtained from the solution of the master equation and the addition of extrinsic noise. For all the curves *K*^*d*^ = 0.1 and 〈*S*_*T*_〉 = 30. The black curve is the pure intrinsic noise case, while the other three correspond to a standard deviation of the Gaussian distribution of *S*_*T*_ equal to 5 (red), 8 (green) and 13 (yellow). (**d**) Examples of probability distribution of *T* in presence of extrinsic noise. The appearance of a bimodal distribution can be modulated by varying the extrinsic noise level. 〈*S*_*T*_〉 = 30, *T*_*T*_ = 40, *K*^*d*^ = 0.1, ${\sigma }_{{S}_{T}}$ assumes the values: 5 (red), 8 (green) and 13 (yellow). (**e**) Plot of the bimodality region (presence of two distinct peaks) for different values of *K*^*d*^ as a function of *T*_*T*_ and extrinsic noise in units of the coefficient of variations ($CV={\sigma }_{{S}_{T}}/\langle {S}_{T}\rangle$). Bimodal distributions are present for parameters inside the areas delimited by the plotted lines. A small *K*^*d*^ and a high noise level favour bimodality. 〈*S*_*T*_〉 = 30, *K*^*d*^ assumes the values: 0.1 (green), 0.2 (light blue) and 0.4 (blue). The size of the step along *T*_*T*_ is Δ*T*_*T*_ = 1, while the size of the step for the extrinsic noise level is Δ*σ*_*T*_ = 0.25 (Δ*CV* = 8⋅10^−3^). For each point defined by these steps, the distribution *P*(*T*) was computed analytically and the number of its maxima was evaluated. Note that the step-like features in the plot are due to the discreteness of *T*_*T*_.

Sequestration dynamics (also known as titrative dynamics) are ubiquitous in nature. Some of the best studied examples concern protein ubiquitination^1^, growth factors signalling^2,3^, gene-expression regulation at the transcriptional and post-transcriptional level, such as transcription factors sequestration^4–6^, the interaction between RNA polymerase and its sigma factors in bacteria^7^, mRNA-miRNA interaction in post-transcriptional gene regulation^8–13^ and bacterial persistence^14^. The general scheme features one (or more) molecular species, the *sequestrant*, that bind to another molecular species, the *target*, to form a complex. In a simple mass-action law scenario, the system displays a threshold-like behaviour of the mean abundance of free targets as a function of the parameters determining its possible maximum amount (e.g. rate of synthesis for open systems or pool size for closed systems) as shown in Fig. 1a and in, *e.g*, refs^6,8,13,15^. This threshold-like behaviour follows from the action of the sequestrant. Intuitively, one can think about the following extreme scenarios. When the total number of the target is lower than the one of the sequestrant most of the target will tend to be bound. However, as the total number of targets begins to outnumber the sequestrant, the average of its free molecules starts to increase more markedly, often in a linear fashion. In the vicinity of the threshold, the average number of free targets moves from the mostly bound, slowly increasing regime, to the unbound, rapidly growing one. Therein, the system is defined to be *ultrasensitive*, meaning that, around the threshold, a small fold change in the amount of total target (input) leads to a larger fold change in the amount of free target (output), corresponding to a Hill coefficient larger than one^6^.

On top of the average behaviour of the systems, one should consider stochastic effects since molecular interactions are known to be probabilistic and to take place in fluctuating environments. While the first source of noise, referred to as intrinsic in the following, has been fairly studied in the past, little is known about the effect of the second one, which we shall call extrinsic^16–18^. This kind of noise may be non-specific, i.e. affects in a similar way many components of a same network and it is often homogenous on a single-cell level but varies from one to another. Sources of extrinsic fluctuations can be variations in the amount of molecular machineries (*e.g*. RNA polymerase, ribosomes, mitochondria), gradients of temperature or chemicals’ concentrations or, more widely, the coupling of the cell to the variability of the external environment. In the present contribution we resort to a minimal model of molecular sequestration and study the system in presence of both intrinsic and extrinsic noise^18,19^. This allows us to focus on two features of sequestration dynamics that have received relatively little attention in the past: the shape of the target probability distribution and the correlations amongst competing targets induced by the presence of the sequestrant. We analytically provide an exact solution to the intrinsic-noise case analysing both targets distributions and correlations in the case of one target and two different targets interacting with one single sequestrant. By adding an extrinsic source of noise, we show how the analytical distributions, previously found to be unimodal, can be reshaped into bimodal in a definite set of parameters. Bimodal distributions are often encountered in gene expression and other biomolecular assays data and are of particular interest as they may indicate the coexistence of two distinct phenotypes, with the modes of the distribution linked to different differentiation states or physiological conditions^20,21^. Inspired by the numerical results of ref.^15^, we first analyse the role of ultrasensitivity close to the threshold as a possible tool to channel extrinsic fluctuations of the parameters of the system into bimodal distributions of free target amount. We find that these bimodal distributions are originated by stochastic effects only, the underlying deterministic system being indeed always monostable. Secondly, we show that the action of the sequestrant is not only responsible for the presence of the threshold which allows to channel noise, but also plays an important role in systems with multiple targets. Indeed, targets result to be effectively correlated by their competing interaction with the same sequestrant and this competition induces a negative correlation. Here we show that, if the amount of sequestrant fluctuates due to extrinsic noise, the above mentioned correlations can, from negative, become positive.

## Results

### Minimal Sequestration Model for noise channelling

#### Sequestration defines the presence of a threshold

Let us consider a simple model consisting of two species, namely *S*, the sequestrant, and *T*, the target. We shall view *S* as a titrating agent that binds *T* and sequesters it in the complex $\overline{TS}$. Here we simply assume a reversible dynamics, where the complex can in turn dissociate, releasing the two species in the environment for further interaction. This first order reaction network is described by the following equation:

1 $$T+S\underset{{k}_{-}}{\overset{{k}_{+}}{\rightleftharpoons }}\overline{TS},$$

where *k*_+_ and *k*_−_ are respectively the binding and unbinding rates. For simplicity, we shall assume in our model the total number of molecules of each species to be conserved, this assumption defining the following conservation laws:

2 $${S}_{T}=S+\overline{TS}=const,$$

3 $${T}_{T}=T+\overline{TS}=const\mathrm{.}$$

In view of these conservation laws, the system has only one free variable, in the following assumed to be *T*. The average behaviour for this model can be studied by considering the associated rate equation for the concentration^6^. We start by writing the steady state solution of the rate equation for the case of two species that bind and unbind, with constant total concentration for each species. The rate equation for the concentration of *T* is:

4 $$\frac{d[T]}{dt}=-\,{\hat{k}}_{+}[T][S]+{\hat{k}}_{-}[\overline{TS}],$$

where ${\hat{k}}_{+}$ and ${\hat{k}}_{-}$ are the reaction rates for the concentrations. In particular ${\hat{k}}_{-}={k}_{-}$ and ${\hat{k}}_{+}={V}_{sys}{k}_{+}$, with *V*_*sys*_ being the typical volume of the system in which reactions occur. By making use of the conservation laws for concentrations that trivially follow from (2) and (3) for constant volumes, the above equation can be easily solved at the steady state giving:

5 $$\mathrm{2[}T]=[{T}_{T}]-[{S}_{T}]-{\hat{K}}^{d}+\sqrt{{([{T}_{T}]-[{S}_{T}]-{\hat{K}}^{d})}^{2}+4{\hat{K}}^{d}[{T}_{T}]},$$

where ${\hat{K}}^{d}\equiv \frac{{k}_{-}}{{k}_{+}{V}_{sys}}$ is the dissociation constant *K*^*d*^ ≡ *k*_−_/*k*_+_ rescaled by the volume. This deterministic approximation can be used to investigate the average behaviour of the number of free target molecules. By rescaling eq. (5) by the volume of the system we obtain:

6 $$2T={T}_{T}-{S}_{T}-{K}^{d}+\sqrt{{({T}_{T}-{S}_{T}-{K}^{d})}^{2}+4{K}^{d}{T}_{T}},$$

which is the steady-state deterministic solution for the amount of free target molecules.

As discussed in^6^, the titrative interaction induces a threshold-like behaviour on the mean number of free molecules, upon the variation of their total amount (see Fig. 1a). The model considered here presents this feature with minimal ingredients, given by the binding and unbinding reactions between *T* and *S* and the conservation laws. Indeed, when the system is in the regime in which the total amount of target molecules is smaller than the sequestrant one, almost all of them are bound in complex and their mean free amount is close to zero. We name this region the *repressed regime*. Conversely, in the regime where the total amount of target molecules is larger than the sequestrant one, their mean number increases linearly with *T*_*T*_, since the number of free molecules of the sequestrant is almost zero. We name this region the *unrepressed regime*. The position of the threshold is then located close to the equimolarity point, *i.e*. where ${T}_{T}\simeq {S}_{T}$. As a direct outcome of this behaviour, the system becomes ultrasensitive in proximity to the threshold. This means that around this point, a small fold-change variation in the total number of target molecules can result in a large fold-change of their mean free amount. In this specific framework, the steepness of the threshold is determined by the dissociation constant *K*^*d*^. It follows that a small *K*^*d*^ implies a large affinity between the molecules, therefore leading to a sharper threshold. In the limit of infinitely large affinity between the sequestrant and the target, the system is continuous at the threshold but displays a discontinuous derivative. In this respect, the usage of the term *ultrasensitivity* depicts a scenario that differs from the ones typical of Goldbeter-Koshland, or cooperative Hill models^22^ which, in the limit of infinite cooperation are discontinuous.

#### Full Master Equation Solution

Very often, in biochemical systems, the numbers of individual molecules at play is low. Fluctuations around the average behaviour (described by the rate equation) become then relevant and, to correctly characterise these systems, it is mandatory to take into account their stochastic nature^17^. In order to do that, we seek the explicit form of the probability distribution of the number of molecules in the system at a given time. Recalling that this model has only one independent variable, let us define *P*(*T*, *t*) as the probability of observing *T* free molecules at time *t*. The dynamics of this probability distribution is Markovian and obeys the following chemical master equation^23,24^:

7 $$\frac{dP(T,t)}{dt}={k}_{+}(T+\mathrm{1)(}S+\mathrm{1)}P(T+1,\,t)+{k}_{-}(\overline{TS}+1)P(T-1,\,t)-[{k}_{+}TS+{k}_{-}\overline{TS}]P(T,t\mathrm{).}$$

Using the conservation laws, eqs (2) and (3), the master equation can be written as:

8 $$\begin{array}{ccc}\frac{dP(T,t)}{dt} & = & {k}_{+}(T+1)({S}_{T}-{T}_{T}+T+1)P(T+1,\,t)\\  &  & +{k}_{-}({T}_{T}-T+1)P(T-1,\,t)\\  &  & -\,[{k}_{+}T({S}_{T}-{T}_{T}+T)+{k}_{-}({T}_{T}-T)]P(T,t).\end{array}$$

We are mostly interested in the long-time behaviour of the system and we will then focus on the steady-state solution $P(T)={\mathrm{lim}}_{t\to \infty }P(T,t)$. Since we have a single independent variable and all reactions are reversible, the detailed balance condition is satisfied. Then, at the steady state, equilibrium is reached and there are no probability flows between the states of the system. An important feature of the system is that the possible range of the number of free *T* depends both on the total number of available target molecules *T*_*T*_ and on the total number of sequestrant molecules *S*_*T*_. Indeed, the minimum number of free *T* can either be *T*_*min*_ = 0 when *S*_*T*_ ≥ *T*_*T*_, or *T*_*min*_ = *T*_*T*_ − *S*_*T*_ when *S*_*T*_ < *T*_*T*_. To obtain the steady-state solution *P*(*T*) to the master equation, one can recursively use the detailed balance condition

9 $${k}_{+}(T+\mathrm{1)(}{S}_{T}-{T}_{T}+T+\mathrm{1)}P(T+\mathrm{1)}={k}_{-}({T}_{T}-T)P(T),$$

which leads to:

10 $$P(T)=\frac{1}{{\mathscr{N}}}{({K}^{d})}^{T}(\begin{array}{c}{T}_{T}\\ T\end{array})\frac{1}{[{S}_{T}-({T}_{T}-T)]!},$$

where the normalisation factor is given by:

11 $${\mathscr{N}}=\sum _{T={T}_{min}}^{{T}_{T}}{({K}^{d})}^{T}(\begin{array}{c}{T}_{T}\\ T\end{array})\frac{1}{[{S}_{T}-({T}_{T}-T)]!}\mathrm{.}$$

The shape of this distribution is mostly determined by the magnitude of the dissociation constant *K*^*d*^ and depends on whether the system is in the repressed (*T*_*T*_ < *S*_*T*_) or unrepressed (*T*_*T*_ > *S*_*T*_) regime. For low values of *K*^*d*^ the distribution of free targets *T* is mostly concentrated around the minimum value *T*_*min*_, which, as mentioned before is 0 in the repressed regime and *T*_*T*_ − *S*_*T*_ in the unrepressed one. Some examples of this probability distribution for different values of the dissociation constant and *T*_*T*_ are reported in Fig. 1b. For the explored parameters range, the target distribution was always unimodal. The various moments of the distributions can be obtained exactly by taking ensemble averages over the probability distribution *P*(*T*) from eq. (10). The mean of *T* can be written in terms of Hypergeometric functions^25,26^ as:

12 $$\{\begin{array}{cccc}\langle T\rangle ={K}^{d}\frac{{T}_{T}}{1-{T}_{T}+{S}_{T}}\frac{{}_{1}{F}_{1}(1-{T}_{T},\,2-{T}_{T}+{S}_{T};-\,{K}^{d})}{{}_{1}{F}_{1}(\,-\,{T}_{T},\,1-{T}_{T}+{S}_{T};-\,{K}^{d})} &  & {T}_{T}\le {S}_{T} & ({T}_{min}=0)\\  & {\rm{i}}{\rm{f}} &  & \\ \langle T\rangle =({T}_{T}-{S}_{T})\frac{{}_{1}{F}_{1}(\,-\,{S}_{T},{T}_{T}\,-\,{S}_{T};-{K}^{d})}{{}_{1}{F}_{1}(\,-\,{S}_{T},1+{T}_{T}-{S}_{T};-{K}^{d})} &  & {T}_{T} > {S}_{T} & ({T}_{min}={T}_{T}-{S}_{T})\end{array}$$

as found in, *e.g*., ref.^27^. Its behaviour is plotted in Fig. 1a confirming how the deterministic analysis of eq. (6) (and ref.^6^), which completely neglected fluctuations, gives an accurate description of the average number of free target molecules.

#### Extrinsic noise and bimodal distributions

In the previous section we focussed on the fluctuations originated by the discrete nature of molecules and the intrinsic randomness of their interactions. By introducing fluctuations in the total number of sequestrant molecules *S*_*T*_, we here investigate the influence of extrinsic noise on the present system. Let us consider different copies of our system with different *S*_*T*_, randomly assigned. This mimics, for instance, the scenario in heterogeneous cell populations randomly sampled. For the sake of simplicity we draw the amount of *S*_*T*_ in each system from a discretised Gaussian *P*(*S*_*T*_), restricted to positive values, with mean 〈*S*_*T*_〉 and standard deviation ${\sigma }_{{S}_{T}}$.

The marginal probability distribution of free target molecules *T* (over the different values of *S*_*T*_), *P*(*T*), is now affected by the fluctuations in *S*_*T*_ and differs from the one given in eq. (10). Nonetheless, it can be expressed by making use of the law of total probability^28^, i.e., via the superposition of the conditional probabilities *P*(*T*|*S*_*T*_) weighed by *P*(*S*_*T*_) as follows:

13 $$P(T)=\sum _{{S}_{T}=0}^{\infty }P(T|{S}_{T})P({S}_{T}),$$

where the expression of the conditional probability *P*(*T*|*S*_*T*_) coincides with the solution of the master equation obtained for a given *S*_*T*_ (Eq. (10)).

Given this scheme, let us now study the consequences of the presence of the extrinsic noise in the system. A first quantity affected by this new source of noise is certainly the average of *T*. As the level of extrinsic noise is increased, the profile as a function of *T*_*T*_ becomes smoother with a less pronounced threshold, while the regions farther away from the threshold are not heavily affected (see Fig. 1c). Indeed, the effects of the extrinsic noise are stronger in the vicinity of the threshold where the system is ultrasensitive. Therein, for a fixed *T*_*T*_, small changes in the number of total sequestrants *S*_*T*_ can make the system transit form the repressed to the unrepressed regime, bearing significant consequences on the shape of the probability distribution. Indeed, stochastically sampling systems above and below the threshold can result in a bimodal distribution. The first peak of such distribution is narrow and located close to the origin *T* = 0. It corresponds to the aggregated contribution of the repressed systems, *i.e*., the ones that picked an *S*_*T*_ larger than *T*_*T*_. The second peak is broader and corresponds to the superposition of the unrepressed systems, each of them with its own mean. We remark that the underlying deterministic system is not bistable and that the observed bimodality is due to the stochastic sampling of the repressed and unrepressed regimes across the threshold. This scenario shows how the threshold feature of the system effectively filters the variability introduced by the extrinsic noise, concentrating the contributions of all the systems below threshold. At this point, one may wonder whether any extrinsic noise would have the same effect on target distributions in any parameter range. The answer is no: bimodal distributions are present only close to the threshold and are favoured by steep thresholds (small *K*^*d*^), which allow sampling between the unrepressed and repressed regimes (see Fig. 1d,e). Furthermore, not all distributions of extrinsic noise may induce bimodals for the target. To this aim, the extrinsic noise is required to have a peaked distribution, sufficiently broad to sample both below and above threshold. This becomes clear by examining the Gaussian case with small variances (see Fig. 1e) and the one of uniformly distributed extrinsic noise considered in the Supplementary Information online. In the latter case, the threshold behaviour concentrates the contribution of the systems below threshold, eventually giving rise to a repressed peak, but no mechanisms could induce the appearance of an unrepressed peak (see Fig. S1 in the SI).

In view of these results, the combination of the threshold-like response produced by the titrative interaction and a suitable extrinsic noise on the total amount of one of the species can be considered a general mechanism to achieve bimodality. This result is also interesting from a pure biological point of view. In biological systems, bimodal distributions of gene expression are particularly interesting as they may indicate the presence of two distinct physiological states. At the level of a cell population, this effect would therefore show heterogeneity in different cell gene expression, as observed in^16^. How this is normally achieved remains unknown but the mechanism here discussed represents a minimal way to obtain bimodal gene expression distributions in systems based on molecular sequestration and subject to extrinsic noise. It is worth noticing that the “static” source of extrinsic noise we consider here is a good approximation of the case of a system with a slow dynamically fluctuating sequestrant. In this case, the system has time to approximately reach a steady state before the amount of sequestrant changes considerably^15^. This timescale separation is thought to be present, for instance, in the mechanism of postranscriptional regulation by microRNAs (short non-coding segments of RNA)^10^. In these systems microRNA molecules bind and sequester coding messenger RNAs and inhibit their translation. The complex formation between microRNA and mRNA takes place on a scale that is much faster than the one of transcription and degradation of microRNA and mRNA. The total number of sequestrant molecules (and the one of target ones) then fluctuates on a slower scale than the sequestration dynamics (note that if one focusses on the complex dynamics without explicitly modelling transcription and degradation, the nature of the fluctuations on the number of the sequestrant molecules becomes extrinsic).

### Sequestration as a tool to couple competing species

#### Intrinsic noise

We consider here a system composed of two molecular species (*T*_1_ and *T*_2_) competing for binding to a third molecule (*S*). The reaction network that defines the minimal model is described by eqs (14) and (15). Both *T*_1_ and *T*_2_ can bind to *S* respectively with rate ${k}_{+}^{1}$ and ${k}_{+}^{2}$ to form the complexes $\overline{{T}_{1}S}$ and $\overline{{T}_{2}S}$ which can then dissociate with rates ${k}_{-}^{1}$ and ${k}_{-}^{2}$, as follows:

14 $${T}_{1}+S\underset{{k}_{-}^{1}}{\overset{{k}_{+}^{1}}{\rightleftharpoons }}\overline{{T}_{1}S},$$

15 $${T}_{2}+S\underset{{k}_{-}^{2}}{\overset{{k}_{+}^{2}}{\rightleftharpoons }}\overline{{T}_{2}S}\mathrm{.}$$

*T*_1_ can be seen to act as a sponge for the common resource *S*, preventing the binding with the competing species *T*_2_ and vice versa. Also in this case, we assume the total amount of each species to be constant so that the following conservation laws hold:

16 $${T}_{1T}={T}_{1}+\overline{{T}_{1}S}=const,$$

17 $${T}_{2T}={T}_{2}+\overline{{T}_{2}S}=const$$

and

18 $${S}_{T}=S+\overline{{T}_{1}S}+\overline{{T}_{2}S}=const\mathrm{.}$$

**Figure 2:**
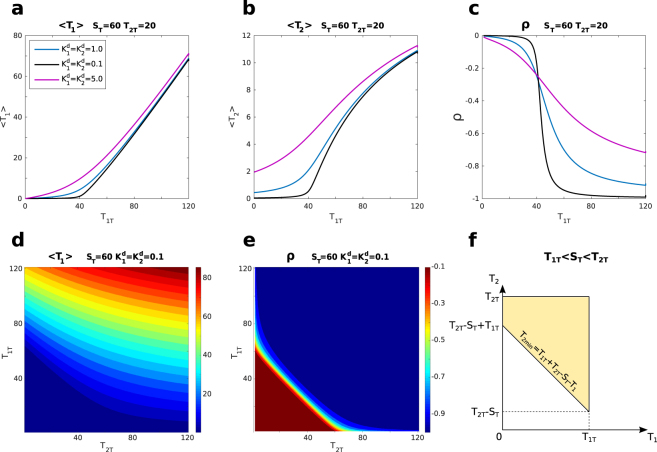
Two targets system. (**a**) Mean value of *T*_1_. (**b**) Mean value of *T*_2_. (**c**) Pearson correlation between *T*_1_ and *T*_2_ as a function of *T*_1*T*_ for a fixed value of *T*_2*T*_ and different values of ${K}_{1}^{d}$ and ${K}_{2}^{d}$. The threshold behaviour of the means and the correlation drop are steeper for lower values of *K*^*d*^. *S*_*T*_ = 60, *T*_2*T*_ = 20. ${K}_{1}^{d}$ and ${K}_{2}^{d}$ are always equal and assume the values: 5.0 (purple), 1.0 (cyan) and 0.1 (black). (**d**) Contour plot of the mean of *T*_1_ as a function of *T*_1*T*_ and *T*_2*T*_. *S*_*T*_ = 60, ${K}_{1}^{d}={K}_{2}^{d}=0.1$. (**e**) Contour plot of the Pearson correlation between *T*_1_ and *T*_2_ as a function of *T*_1*T*_ and *T*_2*T*_. *S*_*T*_ = 60, ${K}_{1}^{d}={K}_{2}^{d}=0.1$. (**f**) Example of phase space for the case *T*_1*T*_ < *S*_*T*_ < *T*_2*T*_.

These conservation laws limit the number of independent variables from 5 to 2. Since there are fewer independent variables than the number of conserved chemical species, the range of values that the number of free molecules of a given species can take is determined by the total amounts of the other species as well as detailed in Table 1. In particular, if the total number of molecules of a species is larger than the one of its titrant, there will always be some free molecule of that species (even if all the available titrant molecules are bound to it) so that its minimal value is greater than 0. When considering two targets, the range of values of free molecules of one target (*e.g. T*_1_) is set by the number of free molecules of the sequestrant, which depends on the amount of complex between the second target and the sequestrant ($\overline{{T}_{2}S}$). For instance, for a fixed amount of free target *T*_2_ the number of free molecules of target 1 ranges between *max*(0, *T*_2*T*_ + *T*_1*T*_ − *S*_*T*_ − *T*_2_) and *T*_1*T*_, as schematically shown in Fig. 2f. In the stochastic description, choosing *T*_1_ and *T*_2_ as independent variables, the model is described by eq. (19), which is the master equation governing the time evolution of the probability distribution of observing *T*_1_ and *T*_2_ free molecules at time *t*, *i.e*.:

19 $$\begin{array}{ccc}\frac{dP({T}_{1},{T}_{2},t)}{dt} & = & (\bar{{T}_{1}S}+1){k}_{-}^{1}P({T}_{1}-1,\,{T}_{2})+({T}_{1}+1)(S+1){k}_{+}^{1}P({T}_{1}+1,\,{T}_{2})\\  &  & +\,(\bar{{T}_{2}S}+1){k}_{-}^{2}P({T}_{1},{T}_{2}-1)+({T}_{2}+1)(S+1){k}_{+}^{2}P({T}_{1},{T}_{2}+1)\\  &  & -\,(\bar{{T}_{1}S}{k}_{-}^{1}+{T}_{1}S{k}_{+}^{1}+\bar{{T}_{2}S}{k}_{-}^{2}+{T}_{2}S{k}_{+}^{2})P({T}_{1},{T}_{2}).\end{array}$$

**Table 1:** Range of allowed values for sequestrant and targets. The table shows the allowed maximum and minimum value for each species, only based on their relative total amounts.

	MIN	MAX
*S*	*max* (0, *S*_*T*_ − *T*_1*T*_ − *T*_2*T*_)	*S*_*T*_
*T*_1_	*max* (0, *T*_1*T*_ − *S*_*T*_)	*T*_1 *T*_
*T*_2_	*max* (0, *T*_2*T*_ − *S*_*T*_)	*T*_2 *T*_

Once again, detailed balance holds and therefore it is possible to derive the equilibrium solution. As presented in the Methods section, the relations obtained imposing detailed balance, together with the conservation laws, can be used to recursively derive the solution of the master equation, eq. (19), which reads:

20 $$P({T}_{1},{T}_{2})=\frac{{({K}_{1}^{d})}^{{T}_{1}}{({K}_{2}^{d})}^{{T}_{2}}}{{{\mathscr{N}}}_{2}}\frac{(\begin{array}{c}{T}_{1T}\\ {T}_{1}\end{array})(\begin{array}{c}{T}_{2T}\\ {T}_{2}\end{array})}{[{S}_{T}+{T}_{1}+{T}_{2}-({T}_{1T}+{T}_{2T})]!},$$

in agreement with the grand canonical distribution for ideal particle mixtures^23^ with the normalization factor given by:

21 $${{\mathscr{N}}}_{2}=\sum _{{T}_{2}=max(0,{T}_{2T}-{S}_{T})}^{{T}_{2T}}\,\sum _{{T}_{1}=max(0,{T}_{2T}+{T}_{1T}-{S}_{T}-{T}_{2})}^{{T}_{1T}}\frac{{({K}_{1}^{d})}^{{T}_{1}}{({K}_{2}^{d})}^{{T}_{2}}(\begin{array}{c}{T}_{1T}\\ {T}_{1}\end{array})(\begin{array}{c}{T}_{2T}\\ {T}_{2}\end{array})}{[{S}_{T}+{T}_{1}+{T}_{2}-({T}_{1T}+{T}_{2T})]!}.$$

#### Thresholds and correlations

As shown in Fig. 2a, the average number of free molecules of target 1, 〈*T*_1_〉, displays a threshold behaviour when plotted as a function of its total number of molecules *T*_1*T*_. The theoretical threshold is again located close to the equimolarity point, *i.e*. where ${T}_{1T}+{T}_{2T}\simeq {S}_{T}$. The average of the other target, 〈*T*_2_〉, shows instead a smooth sigmoidal profile with changing point in the vicinity of the threshold (Fig. 2b). The profiles of both averages are ultrasensitive around the threshold and the strength of the ultrasensitivity is controlled by the dissociation constants (see Fig. 2d). Small values of the dissociation constants mean steeper threshold responses and higher ultrasensitivity.

The common interaction between the targets and the sequestrant effectively correlates them (see e.g. refs^10,11,29^). When several molecules of a target are bound to the sequestrant, the propensity of binding for molecules of the second target is reduced. Additionally, when the total amount of molecules of the two targets globally exceeds the sequestrant, having a high number of one target molecules bound implies having fewer molecules of the other target in a complex. From this, it naturally follows that the two targets are negatively correlated via competition. To show this, we quantify such a correlation by means of the Pearson coefficient of the two targets, defined, as usual, as the ratio between the covariance and the product of the two standard deviations (${\sigma }_{{T}_{1}}$ and ${\sigma }_{{T}_{2}}$) as follows:

22 $$\rho \equiv \frac{{\rm{cov}}({T}_{1},{T}_{2})}{{\sigma }_{{T}_{1}}{\sigma }_{{T}_{2}}},$$

and investigate its dependence on the key parameters in the model. In a later section we will characterise the interaction of the two targets by means of mutual information^30^ (as done in *e.g*. ref.^31^ for a similar setup). In view of the applications of the present framework to biochemical systems, we focus on the dependence of the correlation on the targets abundances which are simpler to control in biological systems than the targets affinity for the sequestrant. Nonetheless, we report in the SI a detailed discussion of the dependence of the correlation on the dissociations constants. In Fig. 2c we plot the Pearson coefficient as a function of the total number of one of the targets (*T*_1*T*_). We observe that the correlation starts close to zero for low target abundances and decreases close to the threshold ${T}_{1T}^{\ast }={S}_{T}-{T}_{2T}$. For low dissociation constants, the Pearson coefficient displays a sigmoidal profile (red curve, Fig. 2c) with the drop located in proximity to the threshold. For high targets abundances, the correlation between the two targets saturates to a value close to −1 (the lower bound of the Pearson coefficient) and this behaviour is maintained also upon varying both targets abundances (Fig. 2e). The shape of the correlation as a function of the total number of one of the targets is affected in a different way by changes of the dissociation constants of the two targets. Keeping *T*_2*T*_ fixed, the steepness of the sigmoidal profile as a function of *T*_1*T*_ is mainly governed by the value of the dissociation constant of target 1, ${K}_{1}^{d}$, and increases with the decrease of the dissociation constant. Instead, ${K}_{2}^{d}$, the dissociation constant of target 2, whose abundance is not changed in the plot, affects the minimal value that the correlation asymptotically reaches as *T*_1*T*_ becomes large (see Fig. S3 in the SI). A lower dissociation constant ${K}_{2}^{d}$ corresponds to a lower value of the minimal correlation (stronger negative correlation). However, this smoothens the correlation drop around the threshold and slightly influences its location. Figure 2e shows the behaviour of the Pearson coefficient when the abundances of both targets are independently changed. No global minimum is found and correlation is stronger (more negative) as the number of target molecules increases.

#### Extrinsic noise

To investigate the effects of external fluctuations, as done for the case of a single target species, we study the behaviour of the two targets system when the number of total sequestrant molecules is allowed to fluctuate between different realisations of the system. The probability distribution of *S*_*T*_ is again assumed to be a discretised Gaussian and the full probability distribution of the system is obtained as a weighed superposition by implementing the law of total probability. Having full analytical control on the system, the behaviour of the correlation in presence of extrinsic noise can be straightforwardly investigated and precisely quantified.

As a result, we observe that fluctuations in the amount of the sequestrant positively correlate the competing targets. This can be understood by considering the fact that the two target species interact with the same pool of sequestrant molecules. Then, fluctuations in the amount of the sequestrant *S*_*T*_ affect in the same way the two target species in each realisation of the extrinsic noise. For instance, if by an extrinsic fluctuation the amount of sequestrant is lower than the average, both target species will have a higher chance of being free from the sequestrant. This effectively induces a positive correlation between the targets, which can counterbalance the negative one due to competition and discussed in the previous section. The negative interference of these two opposite sources of correlation can result in basically uncorrelated systems. However, these two conflicting sources of correlation depend differently on the rates and the abundances. The positive correlation is felt the most in the proximity of the threshold. There, most of the sequestrant is bound and neither of the targets have many free molecules. Then, a change (say a decrease) in the sequestrant abundance directly reflects in a change (increase) in the number of free molecules of both targets. This is no longer the case when there is an excess of one target (e.g. large *T*_1*T*_), for which the sequestrant is bound mostly to *T*_1_ and a change in the sequestrant affects mostly the amount of free *T*_1_ and less the amount of free *T*_2_. When the sequestrant largely outnumbers the targets, changes in its abundance will have little impact on the targets, which will mostly be bound to the sequestrant anyway. From the combination of these effects, correlation takes a non-trivial profile as a function of the abundances and can be positive for sizeable regions of the parameters space.

**Figure 3:**
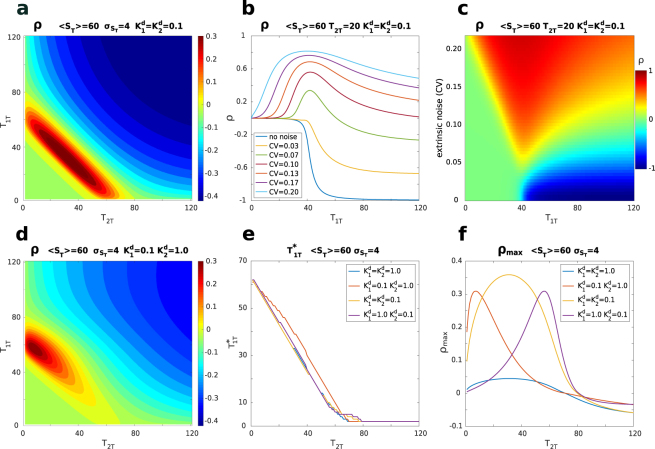
Correlation in presence of extrinsic noise. 〈*S*_*T*_〉 = 60, ${\sigma }_{{S}_{T}}=4$ where not otherwise stated. (**a**,**d**) Contour plots of the correlation as a function of *T*_1*T*_ and *T*_2*T*_ for ${K}_{1}^{d}={K}_{2}^{d}=0.1$ (**a**) and ${K}_{1}^{d}=0.1$, ${K}_{2}^{d}=1.0$ (**d**). (**b**) Correlation as a function of *T*_1*T*_ for different levels of extrinsic noise. The blue line on the bottom corresponds to the pure intrinsic noise case, for the other lines ${\sigma }_{{S}_{T}}$ assumes the values: 2, 4, 6, 8, 10, 12. ${K}_{1}^{d}={K}_{2}^{d}=0.1$, *T*_2*T*_ = 20. (**c**) Contour plot of the correlation as a function of *T*_1*T*_ and of the level of extrinsic noise. ${K}_{1}^{d}={K}_{2}^{d}=0.1$, *T*_2*T*_ = 20. The size of the step along *T*_1*T*_ is Δ*T*_1*T*_ = 1, while the size of the step for the extrinsic noise level is Δ*σ*_*T*_ = 0.25 (Δ*CV* = 4⋅10^−3^). (**e**) Position (in terms of the value of *T*_1*T*_) of the maximum of correlation, for a given *T*_2*T*_, for different values of ${K}_{1}^{d}$ and ${K}_{2}^{d}$. (**f**) Value of the maximum of correlation for a given *T*_2*T*_, for different values of ${K}_{1}^{d}$ and ${K}_{2}^{d}$.

For a more quantitative discussion, we first focus on the correlation profile as a function of the total abundance of target 1, *T*_1*T*_, keeping *T*_2*T*_ fixed. As shown in Fig. 3b,c, a low level of fluctuations on the number of sequestrant molecules opposes the negative correlation induced by competition. When the level of extrinsic noise is increased, the positive correlation induced by the sequestrant fluctuations can counterbalance competition, leading to a correlation close to zero. A further increase of extrinsic noise eventually induces a positive correlation between the targets. Because of the different dependence of the sources of correlation on the system features, the resulting profile displays a correlation maximum in the vicinity of the threshold, where the system is more sensitive to fluctuations of the sequestrant amount. This can also be observed by analysing the two-dimensional (varying both target abundances) profile of correlation shown in Fig. 3a,d. If we focus on slices with a fixed number of *T*_2*T*_ we see that for larger values of *T*_2*T*_ the maximum correlation is reached for lower total amounts of the *T*_1*T*_, for which the system is still in the vicinity of the threshold. This is investigated in Fig. 3e where the abundance of target 1, for which the maximum correlation is attained $({T}_{1T}^{\ast })$, is plotted as a function of the abundance of the second target *T*_2*T*_. When the dissociation constants of the two targets are equal, such position of the maximum decreases linearly (with slope −1) with the increase of the abundance of the second target until the overall number of target molecules (*T*_1*T*_ + *T*_2*T*_) exceeds that of the sequestrant and eventually saturates at low levels. This is more evident for systems with a strong affinity for the sequestrant where the threshold is sharp. The overall trend is preserved when the two targets have different affinities for the sequestrant but the linear decrease is less pronounced when the second target (*T*_2_) has a lower affinity with the sequestrant. When the overall number of targets starts exceeding that of the sequestrant, the correlation decreases and the profile of the location of the maximum depends on the specific dissociation constants. For systems with higher levels of extrinsic noise, such profiles beyond the threshold may be non monotonic, as shown in Fig. S6e in the SI. The value of the maximum of correlation for a given amount of the second target: ${\rho }_{max}({T}_{2T})\equiv \rho ({T}_{1T}^{\ast },{T}_{2T})$ is plotted in Fig. 3f. Such plot can be used to characterise the location and the value of the global maximum of correlation, which is seen when considering the two-dimensional perspective (varying both targets abundances) reported in Fig. 3a,d. Indeed, the maximum of *ρ*_*max*_(*T*_2*T*_) corresponds to the global maximum. As expected, it is located in proximity of the region of equimolarity between targets and sequestrant (${T}_{1T}+{T}_{2T}\simeq {S}_{T}$). The maximizing individual target abundances are set by their respective affinity with the sequestrant: the higher the affinity of a target, the higher its abundance at the maximum. In other words, as shown in Fig. 3f, when the affinities are equal, ${K}_{1}^{d}={K}_{2}^{d}$, the peak is reached for ${T}_{1T}\simeq {T}_{2T}\simeq {S}_{T}\mathrm{/2}$ and when ${K}_{1}^{d} < {K}_{2}^{d}$ the peak moves towards higher values of *T*_1*T*_ and smaller values of *T*_2*T*_. The value of the global maximum is also controlled by the dissociation constants, and takes higher values for lower dissociation constants (higher affinities). Qualitatively similar behaviours are recovered by varying the average amount of sequestrant *S*_*T*_ and the intensity of the noise. We report this in the SI.

### Mutual Information

**Figure 4:**
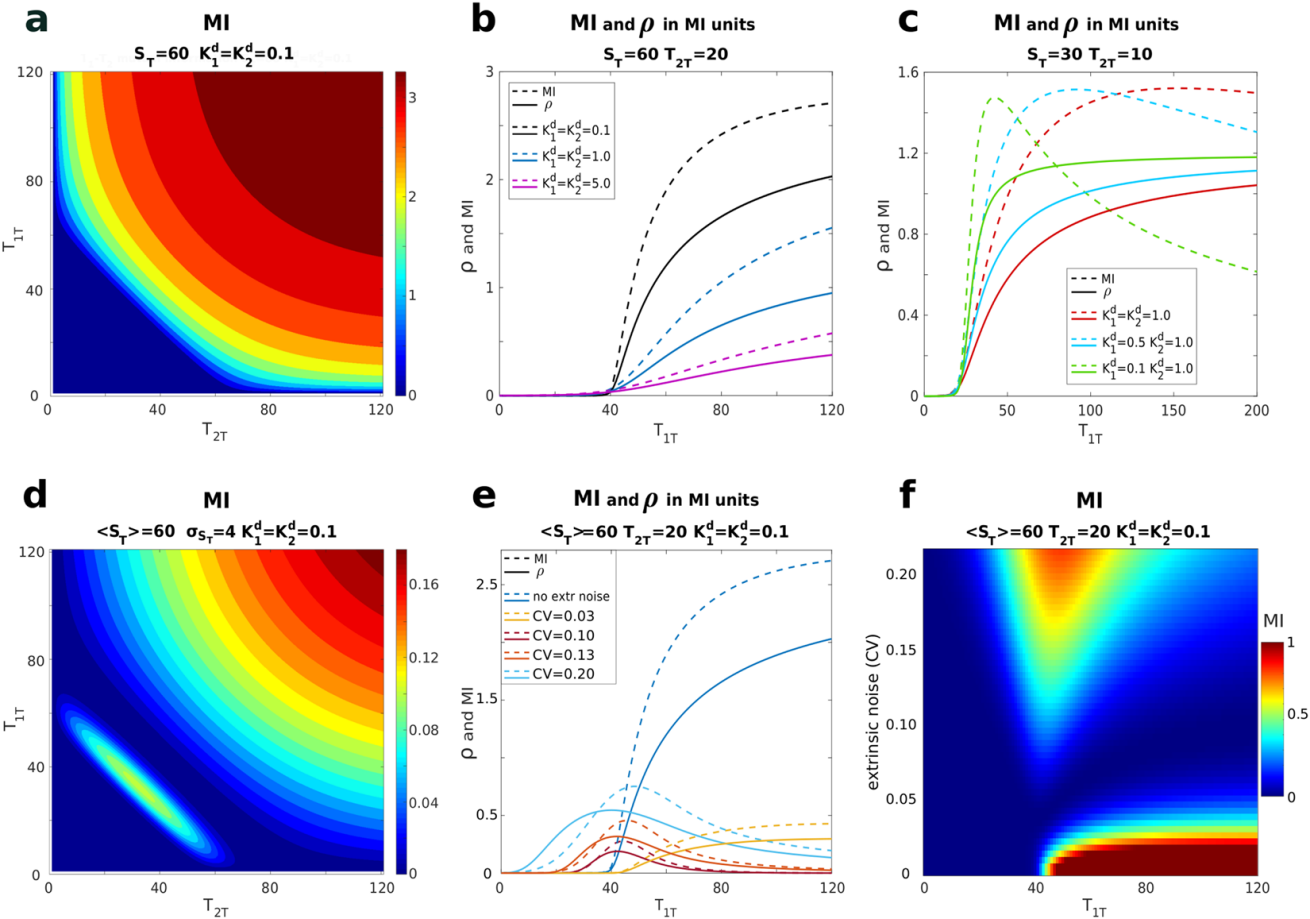
Mutual information (bits) (**a**–**c**) pure intrinsic noise case. (**d**–**f**) Mutual information in presence of extrinsic noise. (**a**) Contour plot of the mutual information between *T*_1_ and *T*_2_ as a function of *T*_1*T*_ and *T*_2*T*_. The parameters are as in Fig. 2e: *S*_*T*_ = 60, ${K}_{1}^{d}={K}_{2}^{d}=0.1$. (**b**) The dashed lines refer to mutual information between *T*_1_ and *T*_2_ as a function of *T*_1*T*_ for a fixed value of *T*_2*T*_ and different values of ${K}_{1}^{d}$ and ${K}_{2}^{d}$. The solid lines are the Pearson correlation expressed in units of $-\frac{1}{2}\,\mathrm{log}[1-{\rho }^{2}]$ as discussed in the text. The values of the parameters correspond to the ones of Fig. 2d: *S*_*T*_ = 60, *T*_2*T*_ = 20. ${K}_{1}^{d}$ and ${K}_{2}^{d}$ are always equal and assume the values: 5.0 (purple), 1.0 (cyan) and 0.1 (black). (**c**) Mutual information and correlation past saturation. Again the dashed lines refer to mutual information and the solid ones are the Pearson correlation expressed in units of $-\frac{1}{2}\,\mathrm{log}[1-{\rho }^{2}]$ the values of the parameters are now different, with *S*_*T*_ = 30, *T*_2*T*_ = 10 and the dissociation constants are reported in the inset. (**d**) Contour plot of the mutual information as a function of *T*_1*T*_ and *T*_2*T*_ for ${K}_{1}^{d}={K}_{2}^{d}=0.1$ in presence of extrinsic noise with 〈*S*_*T*_〉 = 60 and ${\sigma }_{{S}_{T}}=4$ (same parameters as Fig. 3a). (**e**) Mutual information and correlation as a function of *T*_1*T*_ for different levels of extrinsic noise (see the legend). ${K}_{1}^{d}={K}_{2}^{d}=0.1$, *T*_2*T*_ = 20 (same parameters as Fig. 3b) (**f**) Contour plot of the mutual information as a function of *T*_1*T*_ and of the level of extrinsic noise. ${K}_{1}^{d}={K}_{2}^{d}=0.1$, *T*_2*T*_ = 20.

Having access to the explicit expression of the joint probability allows us to characterise the coupling between the competing targets beyond the Pearson coefficient and to study the mutual information between the competing targets. In this setting, mutual information *I*(*T*_1_, *T*_2_) quantifies the amount of information, measured in bits, that could be obtained on one target by measuring the other target. Its definition^30^ reads:

23 $$I({T}_{1},{T}_{2})=\sum _{{T}_{1},\,{T}_{2}}P({T}_{1},{T}_{2})\,\mathrm{log}\,\frac{P({T}_{1},{T}_{2})}{P({T}_{1})P({T}_{2})},$$

where *P*(*T*_1_, *T*_2_) is the joint probability distribution and $P({T}_{1})={\sum }_{{T}_{2}}P({T}_{1},{T}_{2})$ the marginal one. Mutual information is never negative, which implies that both positive and negative correlations result in positive mutual information (there is no distinction between correlated and anti-correlated systems) and, for discrete probabilities, is bounded by the logarithm of the number of states accessible to the system. In Fig. 4a we explore the profile of the mutual information between the targets upon varying both targets’ abundances and in Fig. 4b upon variation of *T*_1*T*_ (in analogy with Fig. 2c,e). The mutual information profile starts at zero and begins to increase in the vicinity of the threshold. As for the Pearson correlation, when plotted as a function of *T*_1*T*_, the dissociation constant ${K}_{1}^{d}$ governs the steepness of the profile, while ${K}_{2}^{d}$ mainly controls the maximum value of mutual information that can be achieved, (see the SI for additional plots). In Fig. 4d–f we show the effect of extrinsic noise on mutual information, in analogy with Fig. 3. The profiles are qualitatively similar to the ones of the Pearson coefficient and offer the same insights. However, mutual information captures further non-linear behaviour in the interaction between two targets. This becomes more evident if we convert correlation in units of $-\frac{1}{2}\,\mathrm{log}[1-{\rho }^{2}]$ where *ρ* is Pearson correlation coefficient, which corresponds to the mutual information that two jointly Gaussian variables of correlation *ρ* would have. This quantity is plotted as full lines in Fig. 4. For the parameters investigated in the previous sections, mutual information takes larger values than the one associated with a Gaussian approximation with the measured Pearson coefficient, showing that the co-dependence between the targets goes beyond the correlation captured by the Pearson coefficient. There is one notable difference between the Pearson correlation and the mutual information, which is seen in the absence of extrinsic noise when one of the target is in large excess (this is not noticeable for the parameters explored in the previous sections) and shown in Fig. 4c. Fixing the abundance of one target and varying the other one (as done in Fig. 4a) correlation is monotonic and reaches a plateau, whereas mutual information displays a maximum when the target starts being in excess. This maximum becomes more peaked as the difference in magnitude of the two dissociation constants increases. The plateau in the Pearson coefficient for an excess target arises from the combined trends of the covariance and the standard deviations. The covariance, similarly to mutual information, is non monotonic and displays a peak. Its decrease after the peak is compensated by the decrease in the standard deviations of the two targets whose marginal distributions get narrower as the system gets more saturated. This discrepancy between the mutual information and the correlation profile can be traced back to the marked non-Gaussianity of the joint target distribution past saturation. In this region the distribution gets strongly peaked, a scenario which has been related to instances of mutual information lower than the one predicted by Gaussian approximations (see *e.g*. ref.^32^). Finally, it is important to note how mutual information (and correlations) are strongly damped by the presence of extrinsic noise as a consequence of the negative interference between the negative correlation due to competition and the positive one induced by fluctuations in the sequestrant.

### Extrinsic noise effects on competitive inhibition kinetics

Let us now explore how the key features we have identified in the minimal model carry over to more complex settings. To this aim, let us consider the case of competitive inhibition in an enzymatic reaction. The system is made of an enzyme that, when free (*T*_*F*_), can bind to a substrate (which we assume to be at fixed concentration *c*_*s*_) and form the active enzyme *T*_*A*_ from which the product is made. The free enzyme can also bind to an inhibitor (*S*) that prevents it from combining with the substrate and becoming active. In the language of the previous sections, the inhibitor plays the role of the sequestrant and the enzyme that of the target. The reaction network that describes this system is defined by

24 $${T}_{F}+S\underset{{k}_{-}}{\overset{{k}_{+}}{\rightleftharpoons }}\overline{TS}$$

25 $$\,{\rm{Substrate}}+{T}_{F}\underset{{k}_{r}}{\overset{{k}_{f}{c}_{s}}{\rightleftharpoons }}{T}_{A}\to {\rm{Product}}+{T}_{F}$$

We consider the case in which both substrate and product concentrations are large so that their fluctuations are negligible and focus on the stochastic dynamics of the enzyme (target) and the inhibitor (sequestrant). Their total amounts are conserved, defining the following conservation laws:

26 $${T}_{T}={T}_{F}+{T}_{A}+\overline{TS}=const,$$

27 $${S}_{T}=S+\overline{TS}=const\mathrm{.}$$

**Figure 5:**
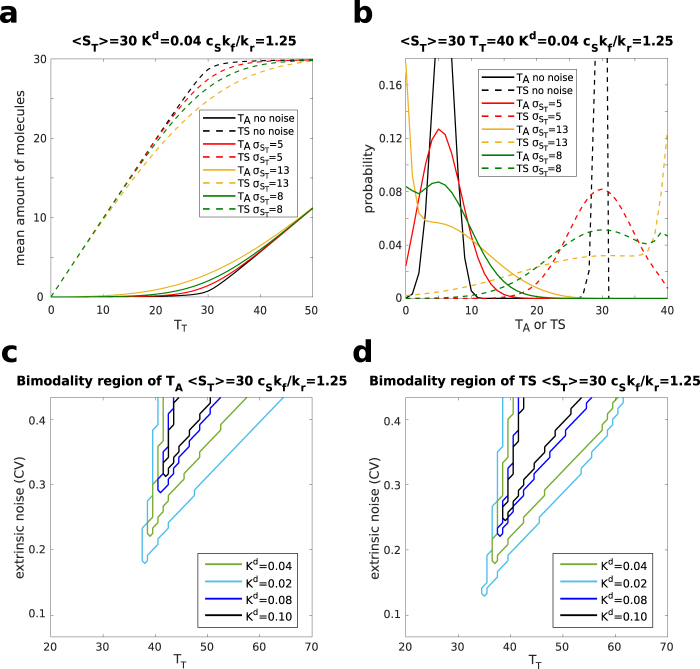
Extrinsic noise effects on competitive inhibition kinetics. (**a**) 〈*T*_*A*_〉 (solid lines) and $\langle \overline{TS}\rangle$ (dashed lines) vs *T*_*T*_ for different levels of extrinsic noise. For all the curves *K*^*d*^ = 0.04, *c*_*S*_*k*_*f*_ /*k*_*r*_ = 1.25 and 〈*S*_*T*_〉 = 30. The black curve is the pure intrinsic noise case, while the other three correspond to a standard deviation of the Gaussian distribution of *S*_*T*_ equal to 5 (red), 8 (green) and 13 (yellow). (**b**) Examples of probability distribution of *T*_*A*_ (solid lines) and $\overline{TS}$ (dashed lines) in presence of extrinsic noise. 〈*S*_*T*_〉 = 30, *T*_*T*_ = 40, *K*^*d*^ = 0.04, *c*_*S*_*k*_*f*_ /*k*_*r*_ = 1.25, ${\sigma }_{{S}_{T}}$ assumes the values: 5 (red), 8 (green) and 13 (yellow). (**c**,**d**) Plots of the bimodality region of the marginal distributions of *T*_*A*_ (**c**) and $\overline{TS}$ (**d**) for different values of *K*^*d*^ as a function of *T*_*T*_ and extrinsic noise. Bimodal distributions are present for parameters inside the areas delimited by the plotted lines. 〈*S*_*T*_〉 = 30, *c*_*S*_*k*_*f*_ /*k*_*r*_ = 1.25, *K*^*d*^ assumes the values: 0.02 (light blue), 0.04 (green), 0.08 (blue) and 0.10 (black). The size of the step along *T*_*T*_ is Δ*T*_*T*_ = 1, while the size of the step for the extrinsic noise level is Δ*σ*_*T*_ = 0.25 (Δ*CV* = 8⋅10^−3^). For each point defined by these steps, the distribution *P*(*T*) was computed analytically and the number of its maxima was evaluated. Note that the step-like features in the plot are due to the discreteness of *T*_*T*_.

As a consequence of the conservation laws, the number of free stochastic variables for this system is reduced to 2. Here, we focus on the active enzyme *T*_*A*_ from which the product is formed and the inhibitor-bound enzyme $\overline{TS}$. For the sake of clarity we consider the case of quasi-equilibrium dynamics in which the product formation reaction is much slower than the others. However, the general case can be addressed with minor modifications since the master equation’s structure and solution is left basically unchanged (see SI). As in the cases considered in the previous sections (and shown in the SI) the steady-state distribution can be analytically derived and the effect of fluctuations in the number of the sequestrant (inhibitor) molecules investigated. We again model the fluctuations by a discretised Gaussian distribution. For comparison with the minimal case, we plot the average behaviour of the active enzyme *T*_*A*_ and the inhibited one $\overline{TS}$ as a function of the total number of enzymes (targets) in Fig. 5a. Both quantities present the threshold-like profile typical of molecular sequestration. As for the minimal case, extrinsic noise affects the shape of the probability distributions and Fig. 5b shows the occurrence of bimodality for both the inhibited complex (sequestrant -target) and the active enzyme (bound to the substrate). However, it is important to note that, due to the presence of an additional chemical reaction, with its inherent stochasticity, the probability distributions are smoother with respect to the minimal model. As a consequence, stronger affinities (lower dissociation constants) are required to ensure robust bimodal phenotypes, especially for the active enzyme (see Fig. 5c,d). In analogy with the minimal model, the distributions can be tuned from being bimodal to unimodal and vice versa by modulating the level of extrinsic noise. The effects of the extrinsic noise level and of the dissociation constant (*K*_*d*_ = *k*_−_/*k*_+_) on bimodality are in qualitative agreement with the minimal model. Indeed, for both *T*_*A*_ and $\overline{TS}$, as extrinsic noise is increased, the range of bimodality over the values of *T*_*T*_ becomes wider. Similarly, a low value of the dissociation constant, i.e. a steeper threshold, favours the presence of bimodality (Fig. 5c,d).

#### Correlations

In the previous sections we showed that two target species competing for the same sequestrant can become positively correlated under the effect of extrinsic noise on the sequestrant. We now analyse the dynamics of two species of enzymes (targets) inhibited by the same sequestrant. The corresponding reaction network is described by the following reactions:

28 $${T}_{1F}+S\underset{{k}_{-}^{1}}{\overset{{k}_{+}^{1}}{\rightleftharpoons }}\overline{{T}_{1}S}$$

29 $${\rm{Substrate}}+{T}_{1F}\underset{{k}_{r}^{1}}{\overset{{k}_{f}^{1}{c}_{s}}{\rightleftharpoons }}{T}_{1A}\to {\rm{Product}}+{T}_{1F}$$

30 $${T}_{2F}+S\underset{{k}_{-}^{2}}{\overset{{k}_{+}^{2}}{\rightleftharpoons }}\overline{{T}_{2}S}$$

31 $${\rm{Substrate}}+{T}_{2F}\underset{{k}_{r}^{2}}{\overset{{k}_{f}^{2}{c}_{s}}{\rightleftharpoons }}{T}_{2A}\to {\rm{Product}}+{T}_{2F}\mathrm{.}$$

**Figure 6:**
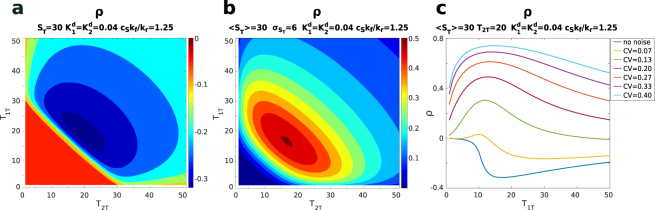
Correlations for competitive inhibition. (**a**) Pure intrinsic noise case: contour plot of the Pearson correlation between the active enzymes *T*_1*A*_ and *T*_2*A*_ as a function of the total number of enzymes *T*_1*T*_ and *T*_2*T*_. *S*_*T*_ = 30, ${K}_{1}^{d}={K}_{2}^{d}=0.04$, ${c}_{S}{k}_{f}^{1}/{k}_{r}^{1}={c}_{S}{k}_{f}^{2}/{k}_{r}^{2}=1.25$. (**b**) Extrinsic noise: contour plot of the Pearson correlation between *T*_1*A*_ and *T*_2*A*_ as a function of *T*_1*T*_ and *T*_2*T*_. 〈*S*_*T*_〉 = 30, ${\sigma }_{{S}_{T}}=6$, ${K}_{1}^{d}={K}_{2}^{d}=0.04$, ${c}_{S}{k}_{f}^{1}/{k}_{r}^{1}={c}_{S}{k}_{f}^{2}/{k}_{r}^{2}=1.25$. (**c**) Correlation between the active enzymes as a function of *T*_1*T*_ for different levels of extrinsic noise. The blue line on the bottom corresponds to the pure intrinsic noise case, for the other lines ${\sigma }_{{S}_{T}}$ assumes the values: 2, 4, 6, 8, 10, 12. 〈*S*_*T*_〉 = 30, *T*_2*T*_ = 20, ${K}_{1}^{d}={K}_{2}^{d}=0.04$, ${c}_{S}{k}_{f}^{1}/{k}_{r}^{1}={c}_{S}{k}_{f}^{2}/{k}_{r}^{2}=1.25$.

Both the unbound targets, *T*_1*F*_ and *T*_2*F*_, can be activated (into *T*_1*A*_ and *T*_2*A*_) by binding to a substrate with rates proportional to the substrate concentration and to their intrinsic activation rates ${k}_{f}^{1}$ and ${k}_{f}^{2}$. For the sake of simplicity, we assume that the substrate is the same for both the enzymes. The active targets *T*_1*A*_ and *T*_2*A*_ can be deactivated with rates ${k}_{r}^{1}$ and ${k}_{r}^{2}$ respectively. The unbound, inactive targets can be sequestered by the inhibitor molecule *S* into the inactive complexes $\overline{{T}_{1}S}$ and $\overline{{T}_{2}S}$, the rates of these reactions are ${k}_{+}^{1}$ and ${k}_{+}^{2}$. Finally, the complexes $\overline{{T}_{1}S}$ and $\overline{{T}_{2}S}$ can dissociate with rates ${k}_{-}^{1}$ and ${k}_{-}^{2}$ respectively. We focus on the correlations that are induced on the active enzymes by the competitive interactions with the inhibitor. In the absence of noise, the two enzymes are negatively correlated, especially in proximity to the threshold (see Fig. 6a). The activation reaction introduces an additional source of stochasticity. As a consequence, the correlations between the active enzymes (*T*_1*a*_ and *T*_2*a*_) are generally lower than the ones between the sequestered, inactive enzymes ($\overline{{T}_{1}S}$ and $\overline{{T}_{2}S}$) and of the ones of the minimal model. This additional layer of stochasticity also affects the profile of the Pearson correlation coefficient, which now displays a minimum around the threshold instead of the plateau shown for the minimal model (see Fig. 2c,e). This can be intuitively understood considering the fact that, in the minimal model, the plateau arose because the standard deviation of the targets roughly followed the behaviour of the covariance. The additional stochasticity related to the activation dynamics is not affected much by the sequestration dynamics, thus the standard deviation keeps growing with the total number of targets not compensating the slow-down of the covariance, giving rise to the minimum.

Let us now turn our attention to the case in which extrinsic noise introduces fluctuations in the number of sequestrant molecules. As in the minimal model, such fluctuations can induce positive correlations between the active target, especially around the threshold (see Fig. 6b,c). Again, since the interaction with the sequestrant is diluted by the presence of an additional stochastic reaction, the correlation tends to be generally lower.

## Discussion

Sequestration models are good approximations of many processes in nature, especially in biological systems. Although fairly studied, this kind of models still show novel features when their stochasticity is addressed. In this contribution we have considered how different sources of noise can affect the properties of a minimal model with sequestration dynamics. We started by analysing again the deterministic version of this system, where, as expected^6^, two species with fixed abundances, here called sequestrant and target, mutually inhibiting each other, give rise to a threshold response. We then moved to a stochastic version taking into account intrinsic noise due to the probabilistic nature of the interactions in the system. Via an exact analytical solution we checked that the threshold behaviour is maintained for the average values of the targets in this context as well. We then made use of the derived set-up to investigate the effects introduced by an extrinsic source of noise, which may be due to fluctuating environments, be they global external surroundings or simply other cell molecular networks interacting with the one under consideration. We have shown that extrinsic fluctuations in the total number of one of the inhibitors (the sequestrant), in combination with the threshold profile may result in a bimodal distribution of the other one (the target). This means that, close to the threshold, the extrinsic variability is channelled by the nonlinear sequestration mechanism into two main outputs corresponding to a repressed and an unrepressed state. This result identifies two minimal ingredients giving rise to bimodal profiles without the need of other regulatory links, i.e., (i) titrative (sequestration) interactions and (ii) extrinsic source of noise.

We have then extended the model to the case of two species competitively binding to a third one. Once again, taking advantage of the simplicity of the minimal model we have derived the analytical solution of the master equations to quantitatively address another key feature of the sequestration dynamics: the correlation induced by the competition for binding to the same sequestrant. We have shown that, without extrinsic fluctuations on the total abundance of the sequestrant, a significant negative correlation is present when the total amount of targets outnumbers the sequestrant. Together with this expected effect of the competition, we have identified non-trivial trends of the correlation in presence of an extrinsic source of noise. Fluctuations in the total sequestrant amount affect in a similar way the free amounts of both targets. As a result, despite the competitive dynamics of the targets, positive correlations are induced by the extrinsic fluctuations of the common sequestrant.

These findings, obtained in a highly simplified description, have highlighted some key features that are likely to occur in more detailed models describing specific biochemical settings. To probe this, we have investigated the more complex case of competitive inhibition in enzymatic kinetics. This system features an additional reaction downstream of the sequestration interaction, which, with its stochasticity, partially dilutes the coupling between competitors, smoothening the distribution profiles and partially hindering the appearance of bimodality. However, we found that, with the due adjustments, extrinsic noise on the inhibitor can induce bimodal distributions and positive correlations between the enzymes. This suggests that the possible constructive role of extrinsic noise we have highlighted in the minimal model may be at play in more complex systems of biological relevance. Models of these systems would be more complicated, including different intermediate steps in the reactions of mediation between sequestrant and target or using saturation functions for production rates. Nonetheless, whenever sequestration dynamics are present, we expect our findings to be of relevance for such complex systems as well, where they may serve as guidelines to identify the most relevant parameters affecting correlations and the presence of bimodal distributions (as for instance in the cases discussed in refs^1–14^). A system for which our results will be of particular importance is the post-transcriptional regulation achieved by short non-coding RNAs interacting with messenger transcripts in eukaryotic cells^10,13,15^.

## Methods

### Solution of the two-targets model

As presented in the main text, from eqs (14) and (15) with the conservation laws (16–18) one can write the master equation that describes the dynamics of the system:

32 $$\begin{array}{ccc}\frac{dP({T}_{1},S,t)}{dt} & = & (\bar{{T}_{1}S}+1){k}_{-}^{1}P({T}_{1}-1,\,S-1)+({T}_{1}+1)(S+1){k}_{+}^{1}P({T}_{1}+1,\,S+1)\\  &  & +\,(\bar{{T}_{2}S}+1){k}_{-}^{2}P({T}_{1},S-1)+({T}_{2}+1)(S+1){k}_{+}^{2}P({T}_{1},S+1)\\  &  & -\,(\bar{{T}_{1}S}{k}_{-}^{1}+{T}_{1}S{k}_{+}^{1}+\bar{{T}_{2}S}{k}_{-}^{2}+{T}_{2}S{k}_{+}^{2})P({T}_{1},S).\end{array}$$

Making use again of the conservation laws (16–18), the master equation can be written in terms of *T*_1_ and *S* only:

33 $$\begin{array}{ccc}\frac{dP({T}_{1},S,t)}{dt} & = & ({T}_{1T}-{T}_{1}+1){k}_{-}^{1}P({T}_{1}-1,S-1)\\  &  & +({T}_{1}+1)(S+1){k}_{+}^{1}P({T}_{1}+1,S+1)\\  &  & +\,({S}_{T}-S-{T}_{1T}+{T}_{1}+1){k}_{-}^{2}P({T}_{1},\,S-1)\\  &  & +\,({T}_{2T}-{S}_{T}+S+{T}_{1T}-{T}_{1}+1)(S+1){k}_{+}^{2}P({T}_{1},S+1)\\  &  & -\,[({T}_{1T}-{T}_{1}){k}_{-}^{1}+{T}_{1}S{k}_{+}^{1}+({S}_{T}-S-{T}_{1T}+{T}_{1}){k}_{-}^{2}\\  &  & +\,({T}_{2T}-{S}_{T}+S+{T}_{1T}-{T}_{1})S{k}_{+}^{2}]P({T}_{1},S).\end{array}$$

In order to find its steady-state solution, we first notice that detailed balance holds. By using detailed balance, the following relations for the steady-state probability are obtained:

34 $${k}_{-}^{1}({T}_{1T}-{T}_{1})P({T}_{1},S)={k}_{+}^{1}({T}_{1}+\mathrm{1)(}S+\mathrm{1)}P({T}_{1}+1,\,S+\mathrm{1),}$$

35 $${k}_{-}^{2}({S}_{T}-{T}_{1T}-S+{T}_{1})P({T}_{1},S)={k}_{+}^{2}(S+\mathrm{1)}({T}_{1T}+{T}_{2T}-{S}_{T}+S-{T}_{1}+1)P({T}_{1},S+\mathrm{1).}$$

Dividing eq. (35) by *P*(*T*_1_), we can write a recursive relation for the probability of having *S* free molecules conditioned on the fact that a generic number *T*_1_ of molecules are free:

36 $$P(S+\mathrm{1|}{T}_{1})=\frac{{k}_{-}^{2}}{{k}_{+}^{2}}\frac{{S}_{T}-{T}_{1T}-S+{T}_{1}}{(S+\mathrm{1)(}{T}_{1T}+{T}_{2T}-{S}_{T}+S-{T}_{1}+\mathrm{1)}}P(S|{T}_{1}\mathrm{).}$$

In this system, as discussed in the main text (and shown for an example in Fig. 2f), the minimal number of free molecules of *S* can either be *S*_*min*_ = 0 if *S*_*T*_ ≤ *T*_1*T*_ + *T*_2*T*_ − *T*_1_, or *S*_*min*_ = *S*_*T*_ − *T*_1*T*_ − *T*_2*T*_ + *T*_1_ when *S*_*T*_ > *T*_1*T*_ + *T*_2*T*_ − *T*_1_. Equation (36) can be used to recursively write the expression of *P*(*S*|*T*_1_) starting from the probability *P*(*S*_*min*_|*T*_1_):

37 $$\begin{array}{rcl}P(S|{T}_{1}) & = & {(\frac{{k}_{-}^{2}}{{k}_{+}^{2}})}^{S-{S}_{min}}\frac{({S}_{T}-{T}_{1T}+{T}_{1}-{S}_{min})!}{({S}_{T}-{T}_{1T}+{T}_{1}-S)!}\frac{{S}_{min}!}{S!}\\  &  & \cdot \,\frac{({S}_{min}+{T}_{1T}+{T}_{2T}-{S}_{T}-{T}_{1})!}{(S+{T}_{1T}+{T}_{2T}-{S}_{T}-{T}_{1})!}P({S}_{min}|{T}_{1})\mathrm{.}\end{array}$$

Writing *P*(*T*_1_, *S*) = *P*(*S*|*T*_1_)*P*(*T*_1_), we can insert eq. (37) in the first relation of detailed balance, eq. (34). Defining ${S}_{min}\equiv {S}_{min}^{\ast }+{T}_{1}$, the obtained relation can be explicited for the marginal probability *P*(*T*_1_ + 1) as follows:

38 $$P({T}_{1}+\mathrm{1)}=\frac{{k}_{-}^{1}}{{k}_{+}^{1}}\frac{{T}_{1T}-{T}_{1}}{({T}_{1}+\mathrm{1)(}{S}_{min}^{\ast }+{T}_{1}+\mathrm{1)}}\frac{P({S}_{min}^{\ast }+{T}_{1}|{T}_{1})}{P({S}_{min}^{\ast }+{T}_{1}+\mathrm{1|}{T}_{1}+1)}P({T}_{1}\mathrm{).}$$

Recursively applying eq. (38), we can write the analytical expression of the marginal probability *P*(*T*_1_):

39 $$P({T}_{1})={(\frac{{k}_{-}^{1}}{{k}_{+}^{1}})}^{{T}_{1}-{T}_{1min}}\frac{({T}_{1T}-{T}_{1min})!{T}_{1min}!({S}_{min}^{\ast }+{T}_{1min})!}{({T}_{1T}-{T}_{1})!{T}_{1}!({S}_{min}^{\ast }+{T}_{1})!}\frac{P({S}_{min}^{\ast }+{T}_{1min}|{T}_{1min})}{P({S}_{min}^{\ast }+{T}_{1}|{T}_{1})}P({T}_{1min}),$$

where *P*(*T*_*min*_) is the probability of having the minimal number allowed of free *T*_1_. *T*_*min*_ is defined by the total amount of *S* and *T*_1_. According to Table 1, the two possible cases can either be *T*_1*min*_ = 0 when *S*_*T*_ ≥ *T*_1*T*_, which means that in principle all the molecules of *T*_1_ can be bound to molecules of *S*, or *T*_1*min*_ = *T*_1*T*_ − *S*_*T*_ when *S*_*T*_ < *T*_1*T*_, meaning that even if all the molecules of *S* are bound to molecules of *T*_1_, *T*_1*min*_ molecules are free. When *T*_1*min*_ > 0 we have that ${S}_{min}^{\ast }+{T}_{1min}={S}_{T}-{T}_{1T}-{T}_{2T}+{T}_{1T}-{S}_{T}=-\,{T}_{2T}\le 0$, then the conditional probability at the numerator in eq. (39) is *P*(0|*T*_1*min*_). Conversely, if *T*_1*min*_ = 0, the conditional probability can either be *P*(0|*T*_1*min*_) or $P({S}_{min}^{\ast }|{T}_{1min})$, depending on the sign of ${S}_{min}^{\ast }$. The probability *P*(*T*_1_, *S*) can be reconstructed by using the definition of joint probability *P*(*T*_1_, *S*) = *P*(*S*|*T*_1_)*P*(*T*_1_), which leads to the following expression:

40 $$P({T}_{1},S)={(\frac{{k}_{-}^{1}}{{k}_{+}^{1}})}^{{T}_{1}}{(\frac{{k}_{-}^{2}}{{k}_{+}^{2}})}^{S-{T}_{1}}\frac{P({T}_{1min},{S}_{min}^{\ast }+{T}_{1min})}{({S}_{T}-{T}_{1T}+{T}_{1}-S)!S!({T}_{1T}+{T}_{2T}-{S}_{T}-{T}_{1}+S)!({T}_{1T}-{T}_{1})!{T}_{1}!},$$

with $P({T}_{1min},{S}_{min}^{\ast }+{T}_{1min})$ given by the normalization of the probability distribution:

41 $$\begin{array}{ccc}P{({T}_{1min},{S}_{min}^{\ast }+{T}_{1min})}^{-1} & = & {\sum }_{{T}_{1}={T}_{1min}}^{{T}_{1T}}{\sum }_{S={S}_{min}^{\ast }+{T}_{1min}}^{{S}_{T}}{(\frac{{k}_{-}^{1}}{{k}_{+}^{1}})}^{{T}_{1}}{(\frac{{k}_{-}^{2}}{{k}_{+}^{2}})}^{S-{T}_{1}}\\  &  & \cdot \,\frac{1}{({S}_{T}-{T}_{1T}+{T}_{1}-S)!S!({T}_{1T}-{T}_{1})!{T}_{1}!}\\  &  & \cdot \,\frac{1}{({T}_{1T}+{T}_{2T}-{S}_{T}-{T}_{1}+S)!}.\end{array}$$

Also for the joint probability if *T*_1*min*_ > 0, then ${S}_{min}^{\ast }+{T}_{1min}\equiv 0$, while when *T*_1*min*_ = 0, the value of ${S}_{min}^{\ast }$ is given by its definition ${S}_{min}^{\ast }\equiv \,{\rm{\max }}({S}_{T}-{T}_{1T}-{T}_{2T},\,0)$.

## Electronic supplementary material

Supplementary Information
