# Supplementary material for: Stochastic sequestration dynamics: a minimal model with extrinsic noise for bimodal distributions and competitors correlation
Source: Sci Rep. 2018 Jul 10;8:10387. doi: 10.1038/s41598-018-28647-9 (PMC6039506; doi:10.1038/s41598-018-28647-9)
Supplement: Supplementary file 1 — Supplementary Information [file 41598_2018_28647_MOESM1_ESM.pdf]

STOCHASTIC SEQUESTRATION DYNAMICS: A MINIMAL MODEL WITH EXTRINSIC NOISE FOR BIMODAL DISTRIBUTIONS AND COMPETITORS CORRELATION - SUPPLEMENTARY INFORMATION

MARCO DEL GIUDICE, CARLA BOSIA, SILVIA GRIGOLON, STEFANO BO

Extrinsic noise with a uniform distribution

Focussing on the system with one target species and a sequestrant, we here investigate the effects of a fluctuating S_T with a uniform distribution $P(S_T)$ defined on the interval $S_{Tmin} \leq S_T \leq S_{Tmax}$:

$$P(S_T) = \frac{1}{S_{Tmax} - S_{Tmin} + 1} \quad . \tag{S1}$$

Mean and variance of the discrete uniform distribution are defined as:

$$\langle S_T \rangle = \frac{S_{Tmax} - S_{Tmin}}{2} \quad , \tag{S2}$$

$$\langle S_T^2 \rangle - \langle S_T \rangle^2 = \frac{(S_{Tmax} - S_{Tmin} + 1)^2 - 1}{12} \quad . \tag{S3}$$

As described in the main text for an S_T with a Gaussian distribution, we derive the free target probability distribution as a weighted superposition of conditional probabilities that are solution of the master equation (eq. (8) in the main text):

$$P(T) = \sum_{S_T=0}^{\infty} P(T|S_T)P(S_T) \quad . \tag{S4}$$

In Fig. S1 we present some examples of $P(T)$ originated from sequestrant probability distributions with mean and variance comparable to the ones of Fig. 1d in the main text.

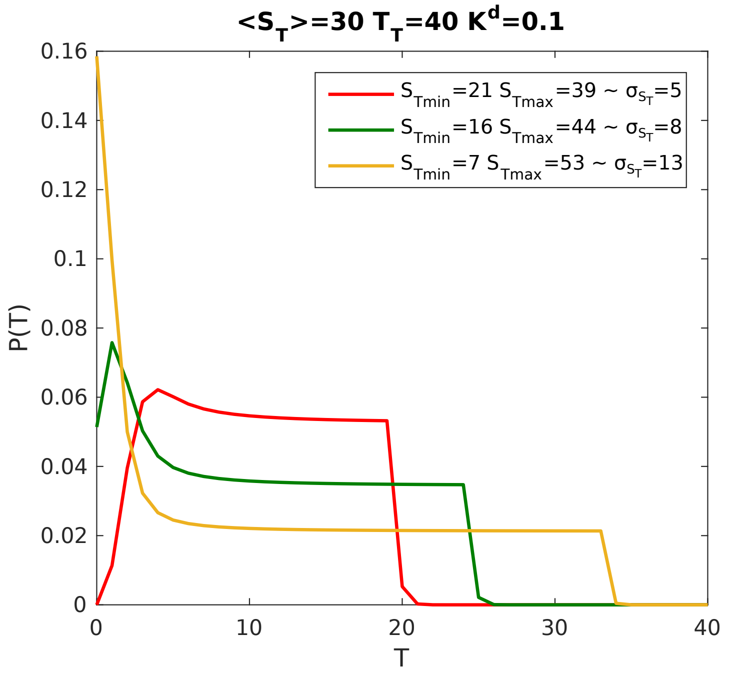

Figure S1. Examples of probability distribution of T in presence of extrinsic noise with uniform distribution. Bimodal distributions cannot be obtained with this kind of extrinsic noise. The values of the parameters correspond to the ones of Fig. 1d in the main text: $\langle S_T \rangle = 30$, $T_T = 40$, $K^d = 0.1$, σ_{S_T} assumes the values: 5 (red), 8 (green) and 13 (yellow).

A uniform extrinsic noise is not able to induce bimodal distributions of the target free amount. Systems that pick a value of S_T below threshold are concentrated by the threshold response into a repressed peak with value of T close to 0. Differently than the Gaussian case, the expressed peak, which corresponded to the peak of the distribution of S_T, cannot be obtained in the uniform case. Indeed, each value of S_T has the same probability and the threshold response does not have any effect in the expressed regime. As a result, the free target probability distribution presents a flat plateau in correspondence to the expressed regime.

In order to obtain bimodal distributions of the free target amount, the extrinsic noise must have a peaked distribution, sufficiently broad to sample both below and above threshold.

Correlation dependence on the total amount of sequestrant

In the main text we kept fixed $\langle S_T \rangle$ and studied the correlation by varying the total amounts of the targets and the level of extrinsic noise, i.e. the variance of the distribution $P(S_T)$. Following this approach, we here investigate the dependence of the correlation on the mean of the extrinsic noise distribution. In the following analysis, the extrinsic noise level is evaluated in terms of the coefficient of variations ($CV = \sigma_{S_T}/\langle S_T \rangle$). Since this quantity depends on the mean of the distribution, when $\langle S_T \rangle$ is varied, the standard deviation is accordingly tuned in order to keep the CV constant.

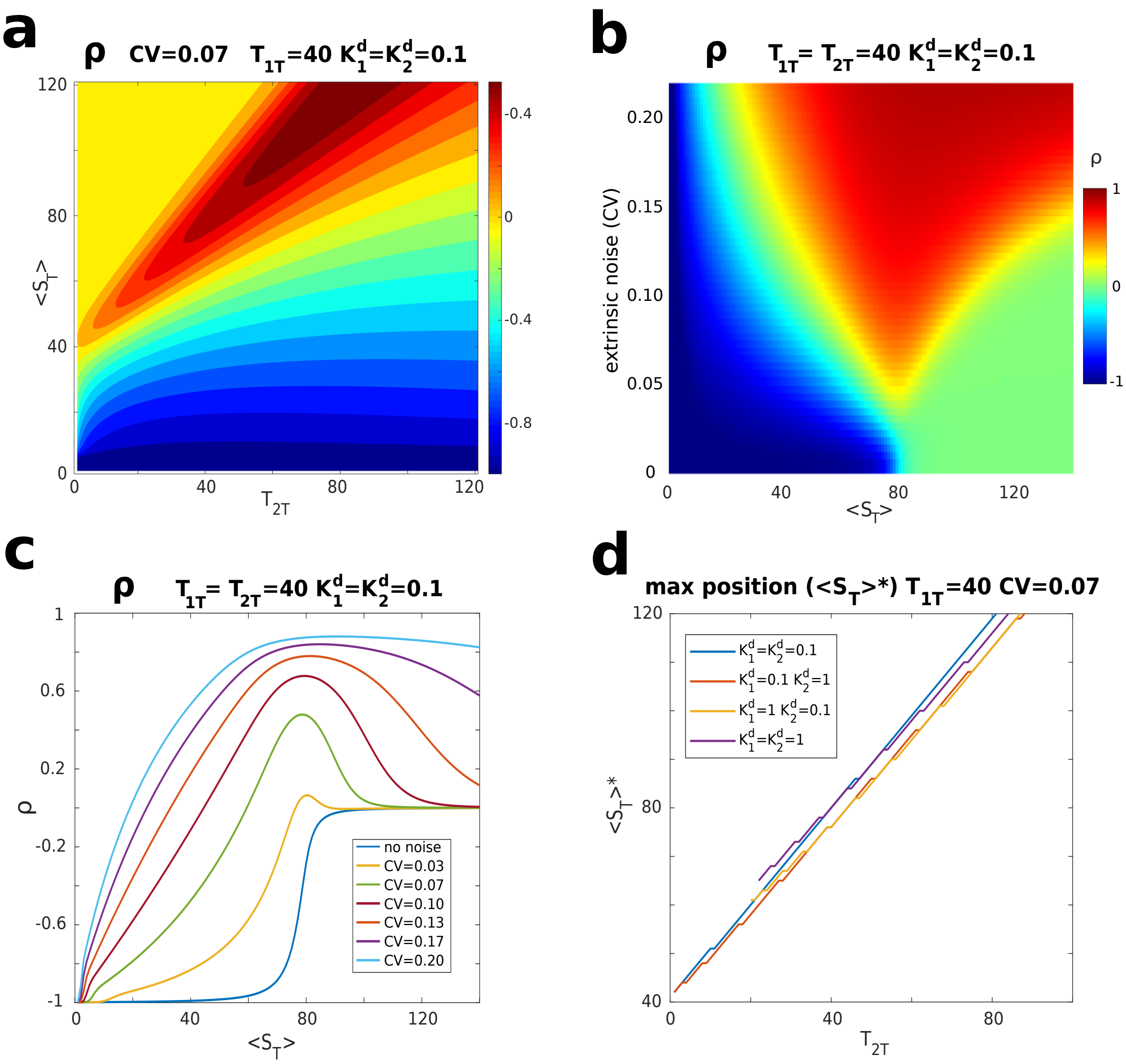

Figure S2. Correlation dependence on $\langle S_T \rangle$ in presence of extrinsic noise. (a) Contour plot of the correlation as a function of $\langle S_T \rangle$ and T_{2T}. $CV = 0.07$, $K_1^d = K_2^d = 0.1$, $T_{1T} = 40$. (b) Contour plot of the correlation as a function of $\langle S_T \rangle$ and of the level of extrinsic noise. $K_1^d = K_2^d = 0.1$, $T_{1T} = T_{2T} = 40$. (c) Correlation as a function of $\langle S_T \rangle$ for different levels of extrinsic noise. The blue line on the bottom corresponds to the pure intrinsic noise case. $K_1^d = K_2^d = 0.1$, $T_{1T} = T_{2T} = 40$. (d) Position (in terms of the value of $\langle S_T \rangle$) of the maximum of correlation, for a given T_{2T}, for different values of K_1^d and K_2^d. $CV = 0.07$, $T_{1T} = 40$. We restrict the plots to cases with non-negligible maximum correlation, $\rho_{max} > 0.05$. As a consequence, the curves where target 1 has low affinity for the sequestrant ($K_1^d = 1$) are truncated for low values of S_T.

The contour plot of the correlation in presence of extrinsic noise as a function of $\langle S_T \rangle$ and T_{2T}, for a fixed value of T_{1T}, is presented in Fig. S2a. As in the analysis of the main text, we observe a region of positive correlation located in proximity to the threshold (the equimolarity point). This behaviour can be better observed in Fig. S2d, where the value of $\langle S_T \rangle$, for which the maximum correlation is attained ($\langle S_T \rangle *$), is plotted as a function of the total amount of the second target T_{2T}. With T_{1T} kept fixed, the position of the correlation maximum increases linearly with T_{2T}, closely following the threshold point.

Fig. S2b,c show the behaviour of the correlation, as a function of $\langle S_T \rangle$, for different levels of extrinsic noise. As in the main text, also in this case we observe that the extrinsic fluctuations oppose the negative correlation induced by competition and eventually lead to a peak of positive correlation which, as described above, is located in proximity to the threshold. The competition-induced negative correlation tends to dominate when the total amount of target molecules is much greater than the sequestrant one, while the global correlation tends to 0 when the sequestrant molecules saturate the system.

Correlation dependence on the dissociation constants

Let us now explore the dependence of the correlation on the two dissociation constants by keeping fixed the total amounts of molecules T_{1T} and T_{2T}. We focus on the case in which the total amount of molecules of one target and its dissociation constant are fixed (*e.g.* K_1^d and T_{1T} fixed). By varying the dissociation constant of the other target (in this case K_2^d), the correlation profile can be non monotonic, displaying a minimum, see figure S3e. We shall refer to the value of the dissociation constant that minimises the Pearson correlation as ${K_2}^{d*}$. As shown in figure S3f, the value of ${K_2}^{d*}$ depends linearly on the fixed dissociation constant of the competing target (K_1^d) and the slope of the dependence is set by the total amount of target molecules T_{2T}. Keeping S_T fixed, the slope decreases as T_{2T} increases and vanishes when $T_{2T} = S_T$. In the regime in which the target outnumbers the sequestrant ($T_{2T} \geq S_T$), the correlation profile is monotonic as a function of the dissociation constant and its lowest value is reached for vanishing K_2^d, regardless of the value of K_1^d. This means that the minimum of the correlation is obtained for the highest affinity between the target and the sequestrant.

Besides the slope of the linear dependence on K_1^d of the minimum position ${K_2}^{d*}$, the total amount of target molecules T_{2T} influences also the offset. When the total number of targets exceeds that of the sequestrant ($T_{1T} + T_{2T} \leq S_T$) at $K_1^d = 0$, the correlation is minimised by a finite value of ${K_2}^{d*}$. Conversely, for $T_{1T} + T_{2T} > S_T$ the offset disappears and a vanishing K_1^d corresponds to a vanishing ${K_2}^{d*}$. In this region of the parameters, the minimum of the correlation is reached for the highest affinity of both the targets. The existence of the offset in the position of ${K_2}^{d*}$ as a function of K_1^d indicates the presence of two sub-regimes characterised by a different joint dependence on the two dissociation constants K_1^d and K_2^d. What determines these regimes is the total number of target molecules in the system $T_{1T} + T_{2T}$, compared to the total number of sequestrant molecules S_T. When the sequestrant is more abundant than the two targets ($T_{1T} + T_{2T} \leq S_T$) there are always some free molecules of the sequestrant and both the targets are in the repressed state. Nonetheless, these two targets can be correlated and their correlation presents a global minimum as a function of the two dissociation constants, see figure S3 (a, b). The presence of a global minimum for finite values of the dissociation constants justifies the existence of the offset in the 1-dimensional plots. For systems in which the number of target molecules is small, the global minimum can be located at relatively large values of the dissociation constants, corresponding to a condition of weak interaction between the targets and the sequestrant.

As an example, let us consider again figure S3. In the first regime, for the case in which $T_{1T} = T_{2T} = 15$ and $S_T = 30$, we see that the minimum of the correlation is present and is obtained for values of the dissociation constants for which the average number of free target is low $\langle T_1 \rangle = \langle T_2 \rangle \simeq 2$ and $\langle S \rangle \simeq 4$. Moving into the second regime, where $T_{1T} + T_{2T} \geq S_T$, we see that there is no global minimum and correlation is lower for lower values of the dissociation constants. Nonetheless, even when the global minimum is absent, considering slices of the contour plot (*e.g.* for K_1^d fixed) the correlation still presents a minimum if $T_{2T} < S_T$. Finally, when $T_{2T} > S_T$, the local minimum in the 1-D plot of the correlation as a function of K_2^d is lost and the minimal value of correlation is reached for vanishing K_2^d.

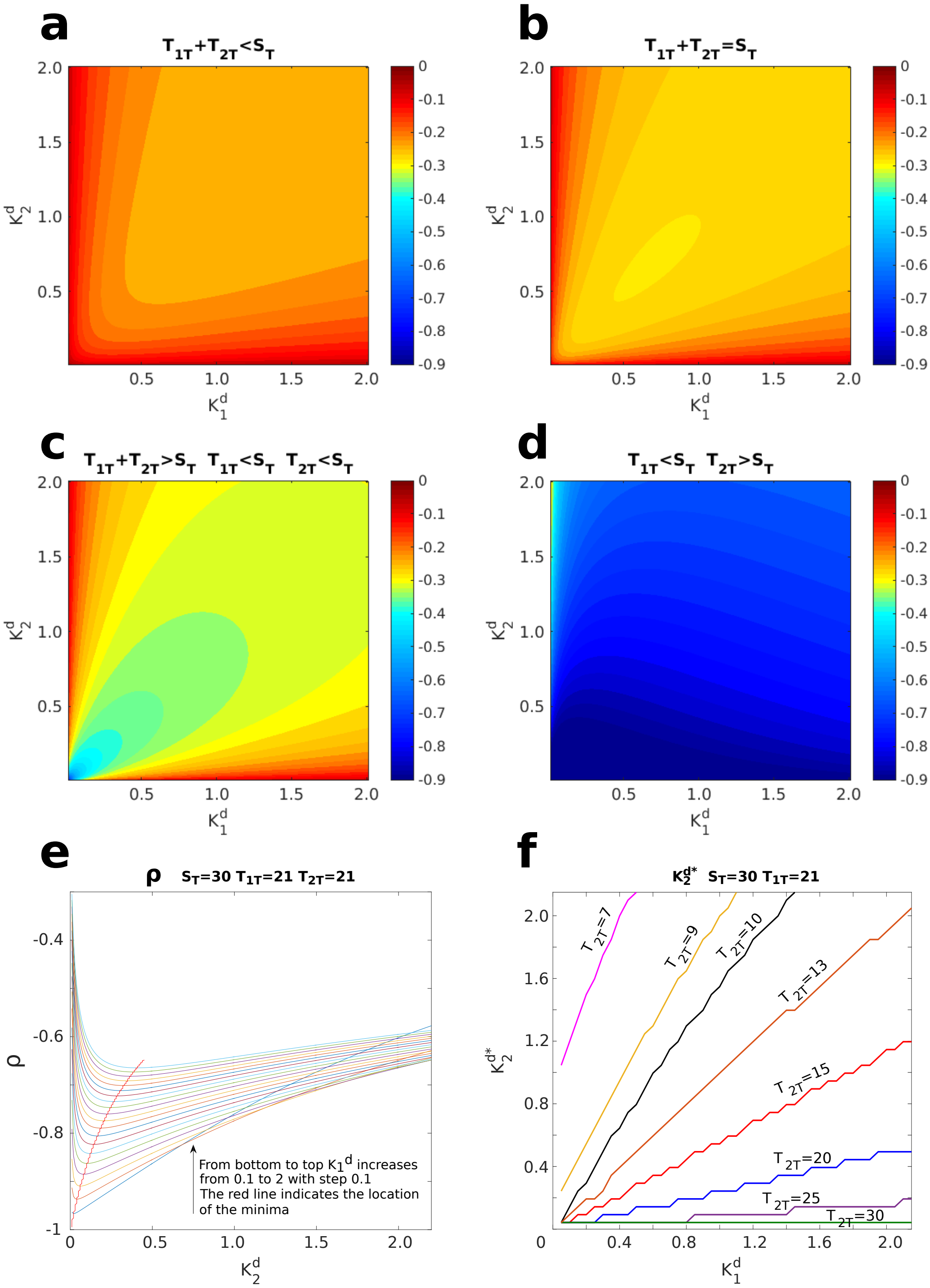

FIGURE S3. Correlation as a function of the dissociation constants. (a-d) Correlation as a function of K_1^d and K_2^d for different values of T_{1T} and T_{2T} and a given value of $S_T = 30$. In (a) $T_{1T} = 15$ and $T_{2T} = 14$, in (b) $T_{1T} = 15$ and $T_{2T} = 15$, in (c) $T_{1T} = 15$ and $T_{2T} = 16$, in (d) $T_{1T} = 15$ and $T_{2T} = 31$. (e) Examples of correlation profile as a function of K_2^d for different fixed values of K_1^d. The red line indicates the location of the minima. $S_T = 30$, $T_{1T} = 21$, $T_{2T} = 21$. (f) Position of the minimum K_2^{d*} as a function of K_1^d for different values of T_{2T} (see legend). $S_T = 30$, $T_{1T} = 21$.

We here report a plot analogous to Fig. 3 in the main text but for higher levels of extrinsic noise (a standard deviation of 6 instead of 4) and higher dissociation constants. The main qualitative difference is the behaviour is contained in Fig. S4e where the abundance of target 1, for which the maximum correlation is attained (T^*_{1T}), is plotted as a function of the abundance of the second target T_{2T}. When the dissociation constants of the two targets are different the linear decrease of the maximum position may have a slope larger than -1. The profile after the overall number of target molecules exceeds that of the sequestrant depends more markedly on the specific dissociation constants and is, in general, non monotonic. The other features are qualitatively unchanged.

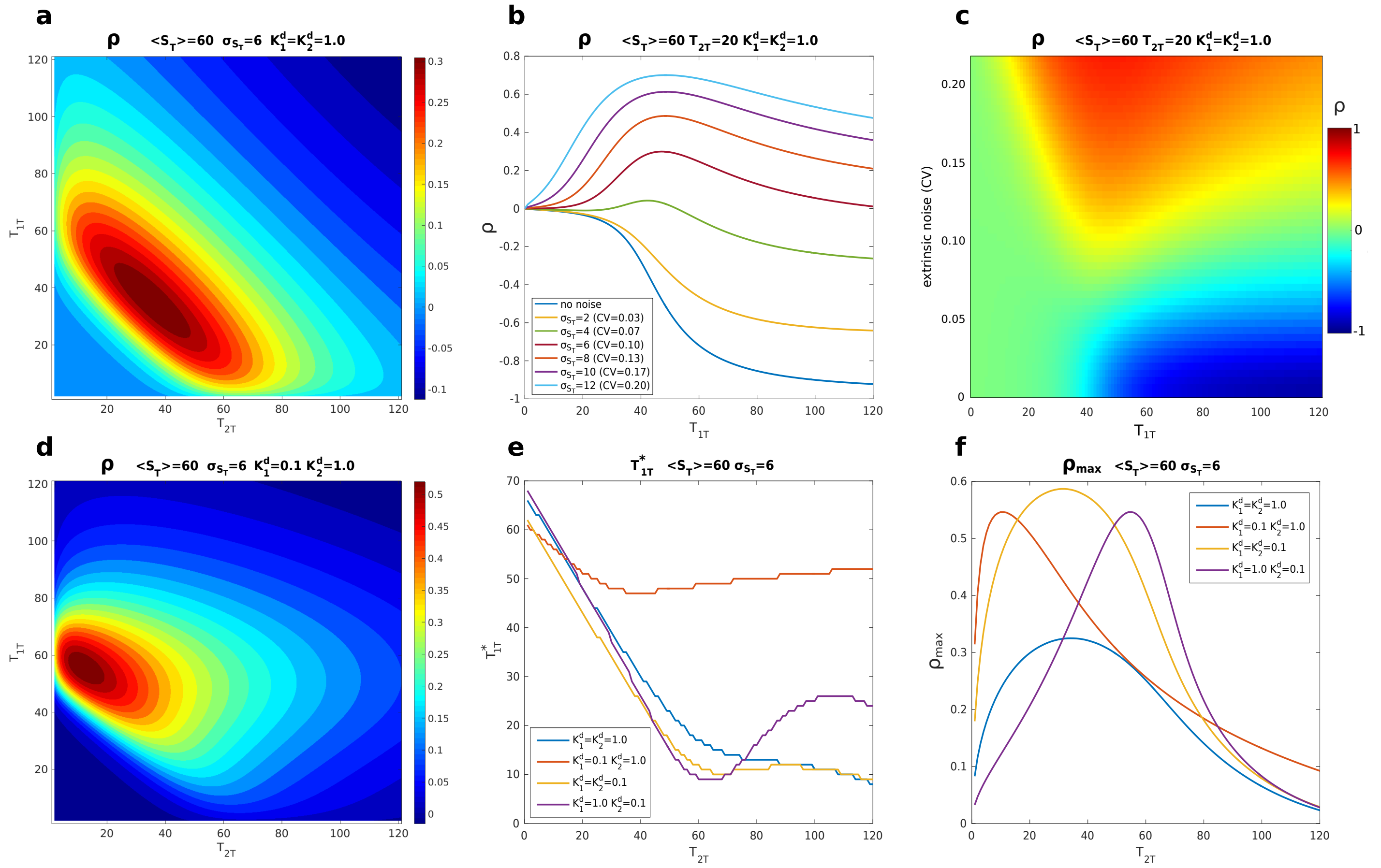

FIGURE S4. Correlation in presence of extrinsic noise. $\langle S_T \rangle = 60$, $\sigma_{S_T} = 6$ where not otherwise stated. (a, d) Contour plots of the Pearson correlation coefficient as a function of T_{1T} and T_{2T} for $K^d_1 = K^d_2 = 1$ (a) and $K^d_1 = 1$, $K^d_2 = 0.1$ (d). (b) Correlation as a function of T_{1T} for different levels of extrinsic noise. The blue line on the bottom corresponds to the pure intrinsic noise case, for the other lines σ_{S_T} assumes the values: 2, 4, 6, 8, 10, 12. $K^d_1 = K^d_2 = 1$, $T_{2T} = 20$. (c) Contour plot of the correlation as a function of T_{1T} and of the level of extrinsic noise. $K^d_1 = K^d_2 = 1$, $T_{2T} = 20$. The size of the step along T_{1T} is $\Delta T_{1T} = 1$, while the size of the step for the extrinsic noise level is $\Delta\sigma_T = 0.25$ ($\Delta CV = 4 \cdot 10^{-3}$). (e) Position (in terms of the value of T_{1T}) of the maximum of correlation, for a given T_{2T}, for different values of K^d_1 and K^d_2. (f) Value of the maximum of correlation for a given T_{2T}, for different values of K^d_1 and K^d_2.

In this section we provide additional plots for parameters that are not show in the main text. As presented in the main text and shown in Fig. S5, the mutual information profile starts at zero and sharply increases in the vicinity to the theoretical threshold. As for the Pearson correlation, when plotted as a function of T_{1T}, the dissociation constant K_1^d governs the steepness of the profile, while K_2^d mainly controls the maximum value of mutual information that can be achieved, see Fig. S5.

In presence of extrinsic noise, the mutual information conveys qualitatively the same message as the Pearson correlation. To compare directly the two quantities it is useful to express correlation in units of $-\frac{1}{2}\log[1-\rho^2]$ where ρ is Pearson correlation coefficient (*i.e.* the mutual information that two jointly Gaussian variables of correlation ρ would have). Fig. S6 shows how the profiles of the two quantities display a qualitatively similar behaviour.

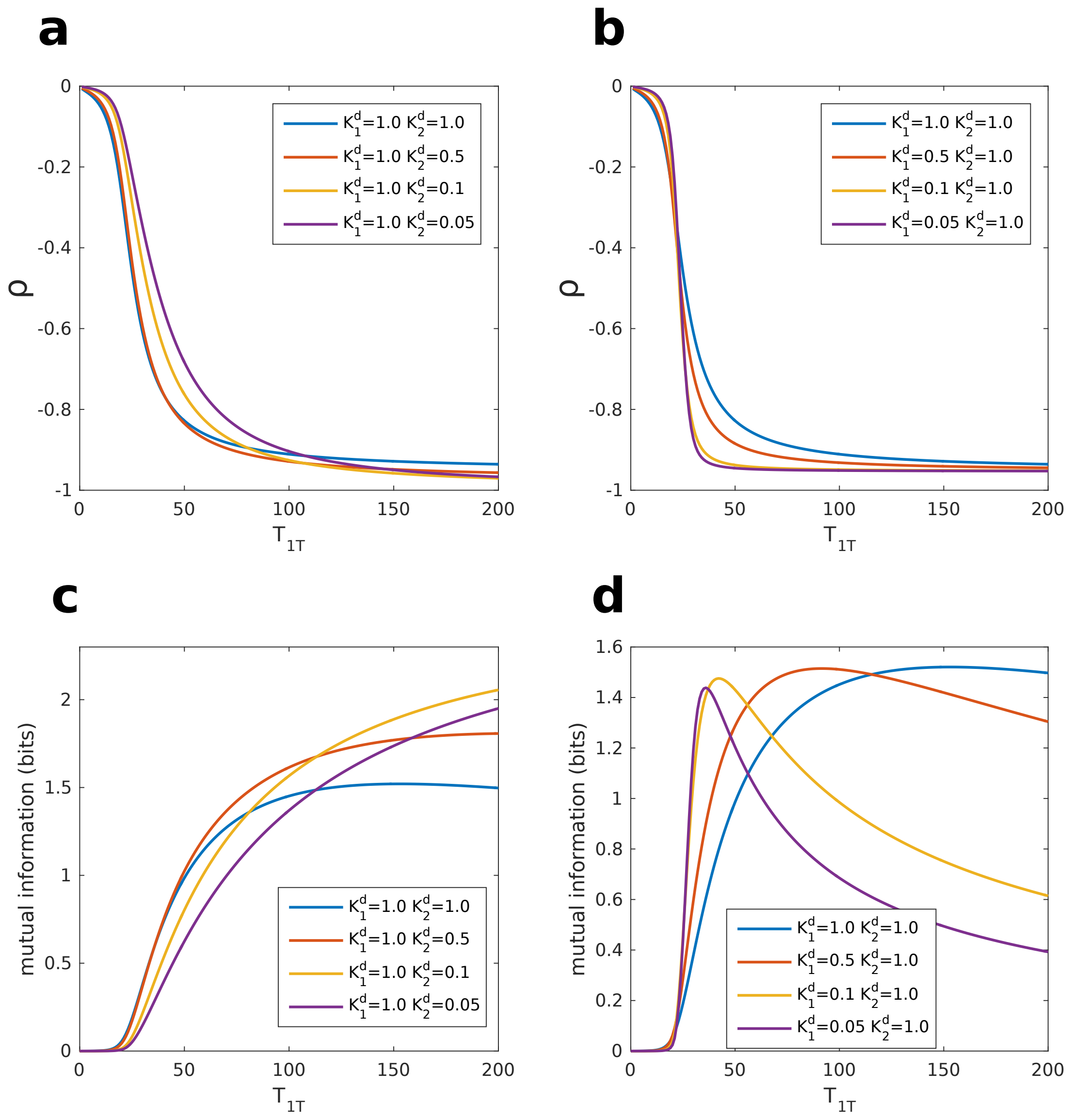

Figure S5. Pearson correlation (a,b) and mutual information (bits) (c,d) as a function of T_{1T}, for different values of the dissociation constants (see legends). $S_T = 30$, $T_{2T} = 10$.

Competitive inhibition

We here present in detail the model of competitive inhibition kinetics that is discussed in the main text. This system is a model of enzymatic kinetics based on the interaction between an enzyme and its inhibitor. The inhibitor plays the role of the sequestrant and the enzyme is the target.

A free molecule of target, T_F, can become active by binding to a substrate which is assumed to be at fixed concentration. The activation of the target occurs with a rate that depends on the intrinsic activation rate k_f and on the concentration of substrate c_S. The active target molecule, T_A, can be deactivated with rate k_r becoming T_F. The free molecule of target

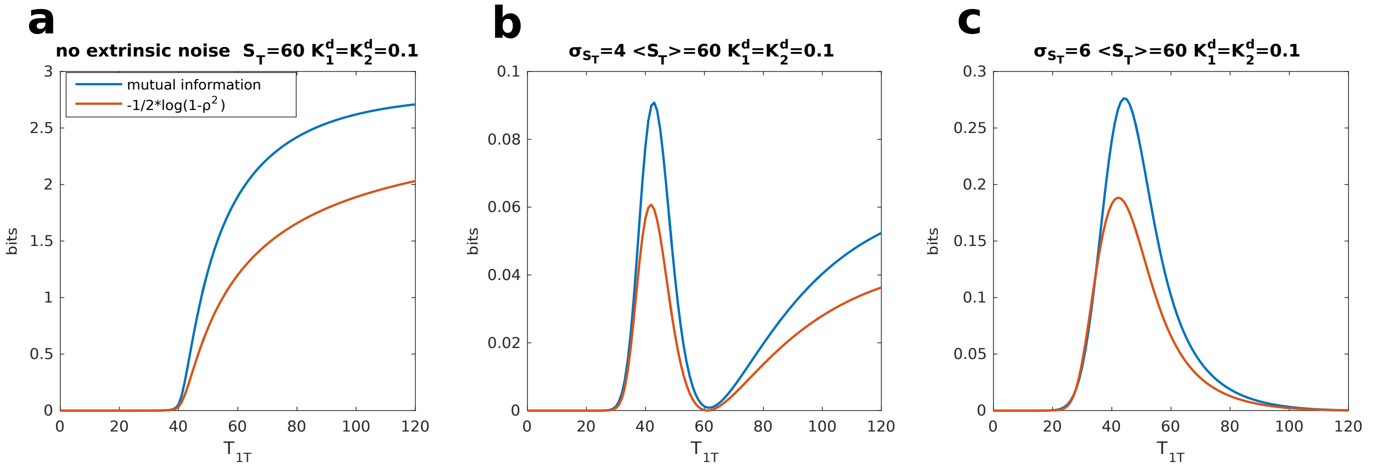

FIGURE S6. Comparison between the mutual information as a function of T_{1T} and the correlation in units of $(-1/2\log(1-\rho^2))$ for different levels of extrinsic noise: no extrinsic noise (a), $\sigma_{S_T} = 4$ (b), $\sigma_{S_T} = 6$ (c). $K_1^d = K_2^d = 0.1$, $T_{2T} = 20$.

can also be bound by a free molecule of sequestrant, S, forming the complex $\overline{TS}$ with rate k_+. The complex can in turn dissociate with rate k_-, returning a free molecule of target and one of sequestrant. The reaction network is then:

$$T_F + S \underset{k_-}{\overset{k_+}{\rightleftharpoons}} \overline{TS} \quad ,$$

$$\text{Substrate} + T_F \underset{k_r}{\overset{k_f c_s}{\rightleftharpoons}} T_A \xrightarrow{k_p} \text{Product} + T_F \quad .$$

We assume that the concentrations of substrate and product are large, we then neglect their fluctuations and study the stochastic dynamics of target and sequestrant. In addition to the reactions above, we assume that the total amounts of target and sequestrant molecules are conserved, defining then the following conservation laws:

(S5) $$T_T = T_F + T_A + \overline{TS} = const \quad ,$$

(S6) $$S_T = S + \overline{TS} = const \quad .$$

As a consequence of the conservation laws, the number of free variables for this system is reduced to 2. We here focus on T_A and $\overline{TS}$. The master equation describing the time evolution of their joint probability distribution reads:

(S7) $$\begin{aligned}\frac{dP(T_A,\overline{TS},t)}{dt} &= k_f c_s(T_T - T_A + 1 - \overline{TS})P(T_A - 1,\overline{TS},t) + k_r(T_A+1)P(T_A+1,\overline{TS},t) + \\ &+ k_+(T_T - T_A - \overline{TS} + 1)(S_T - \overline{TS} + 1)P(T_A,\overline{TS}-1,t) + k_-(\overline{TS}+1)P(T_A,\overline{TS}+1,t) + \\ &- [k_f c_s(T_T - T_A - \overline{TS}) + k_r T_A + k_+(T_T - T_A - \overline{TS})(S_T - \overline{TS}) + k_-\overline{TS}]P(T_A,\overline{TS},t) \quad .\end{aligned}$$

We focus on the case of quasi-equilibrium dynamics in which the reaction of product formation is much slower than the others, which means neglecting the product forming reaction. The master equation for the general case has a very similar structure, with the main difference that there is now an additional reaction converting an active enzyme (bound to the substrate) T_A to an inactive and free one T_F. For the general case, then, the rate k_r should be replaced by $k_r + k_p$.

The steady-state solution of the master equation can be written recalling the grand canonical distribution for ideal particle mixtures [2] and reads:

(S8) $$P(T_A,\overline{TS}) = \frac{1}{N}(K^d)^{-\overline{TS}}\left(\frac{k_f c_s}{k_r}\right)^{T_A}\frac{1}{\overline{TS}!(S_T-\overline{TS})!T_A!(T_T-T_A-\overline{TS})!} \quad ,$$

where $K^d = k_-/k_+$ and

(S9) $$N = \sum_{T_A=0}^{T_T}\sum_{\overline{TS}=0}^{min(T_T-T_A,S_T)}\frac{1}{N}(K^d)^{-\overline{TS}}\left(\frac{k_f c_s}{k_r}\right)^{T_A}\frac{1}{\overline{TS}!(S_T-\overline{TS})!T_A!(T_T-T_A-\overline{TS})!} \quad .$$

Since we are interested in the effects of extrinsic noise on this model of competitive inhibition, we again assume that the total amount of sequestrant molecules is a fluctuating quantity described by a discretised Gaussian distribution $P(S_T)$. The analytic equilibrium solution in presence of extrinsic noise can be obtained as a weighted superposition of conditional probabilities:

$$P(T_A,\overline{TS}) = \sum_{S_T=0}^{\infty} P(T_A,\overline{TS}|S_T)P(S_T) \quad , \tag{S10}$$

where $P(T_A,\overline{TS}|S_T)$ are solutions of the master equation (S7) with a given value of S_T.

Competitive inhibition model with two enzymes. Our aim is now to study the correlations induced in this system by competition and extrinsic noise. To do that, we extend the model above by adding a second enzyme that can be activated by binding to a substrate and that is sequestered by the same inhibitor of the previous one. For simplicity we assume that the two enzymes bind to the same substrate. The reaction network for this extended model is:

$$T_{1F} + S \underset{k_-^1}{\overset{k_+^1}{\rightleftharpoons}} \overline{T_1S} \quad ,$$

$$\text{Substrate} + T_{1F} \underset{k_r^1}{\overset{k_f^1 c_s}{\rightleftharpoons}} T_{1A} \rightarrow \text{Product} + T_{1F} \quad ,$$

$$T_{2F} + S \underset{k_-^2}{\overset{k_+^2}{\rightleftharpoons}} \overline{T_2S} \quad ,$$

$$\text{Substrate} + T_{2F} \underset{k_r^2}{\overset{k_f^2 c_s}{\rightleftharpoons}} T_{2A} \rightarrow \text{Product} + T_{2F} \quad .$$

As in the other models, we assume that the total amounts of sequestrant and targets molecules are conserved, obtaining the following conservation laws:

$$T_{1T} = T_{1F} + T_{1A} + \overline{T_1S} = const \quad , \tag{S11}$$
$$T_{2T} = T_{2F} + T_{2A} + \overline{T_2S} = const \quad , \tag{S12}$$
$$S_T = S + \overline{T_1S} + \overline{T_2S} = const \quad . \tag{S13}$$

These conservation laws reduce the number of free variables to 4, from now on we will focus on active targets and complexes. To use a lighter notation, we define the probability of observing $\overline{n} = (n_{T_{1A}}, n_{T_{2A}}, n_{\overline{T_1S}}, n_{\overline{T_2S}}) \equiv (T_{1A}, T_{2A}, \overline{T_1S}, \overline{T_2S})$ molecules at time t as: $P(\overline{n},t) \equiv P(T_{1A}, T_{2A}, \overline{T_1S}, \overline{T_2S}, t)$. The master equation describing the time evolution of this probability distribution reads:

$$\begin{aligned}
\frac{dP(\overline{n},t)}{dt} &= k_f^1 c_s(T_{1T} - T_{1A} + 1 - \overline{T_1S})P(\overline{n}_{T_{1A}} - 1, t) + k_r^1(T_{1A}+1)P(\overline{n}_{T_{1A}}+1,t) + \\
&+ k_+^1(T_{1T} - T_{1A} - \overline{T_1S} + 1)(S_T - \overline{T_1S} - \overline{T_2S} + 1)P(n_{\overline{T_1S}} - 1, t) + k_-^1(\overline{T_1S}+1)P(n_{\overline{T_1S}}+1,t) + \\
&- [k_f^1 c_s(T_{1T} - T_{1A} - \overline{T_1S}) + k_r^1 T_{1A} + k_+^1(T_{1T} - T_{1A} - \overline{T_1S})(S_T - \overline{T_1S} - \overline{T_2S}) + k_-^1\overline{T_1S}]P(\overline{n},t) + \\
&+ k_f^2 c_s(T_{2T} - T_{2A} + 1 - \overline{T_2S})P(\overline{n}_{T_{2A}} - 1, t) + k_r^2(T_{2A}+1)P(\overline{n}_{T_{2A}}+1,t) + \\
&+ k_+^2(T_{2T} - T_{2A} - \overline{T_2S} + 1)(S_T - \overline{T_1S} - \overline{T_2S} + 1)P(n_{\overline{T_2S}} - 1, t) + k_-^2(\overline{T_2S}+1)P(n_{\overline{T_2S}}+1,t) + \\
&- [k_f^2 c_s(T_{2T} - T_{2A} - \overline{T_2S}) + k_r^2 T_{2A} + k_+^2(T_{2T} - T_{2A} - \overline{T_2S})(S_T - \overline{T_1S} - \overline{T_2S}) + k_-^2\overline{T_2S}]P(\overline{n},t) \quad .
\end{aligned} \tag{S14}$$

The analytic steady-state solution of the master equation above is [2]:

(S15)
$$P(T_{1A}, T_{2A}, \overline{T_1S}, \overline{T_2S}) = \frac{1}{N}\left(\frac{k_f^1 c_s}{k_r^1}\right)^{T_{1A}}\left(\frac{k_f^2 c_s}{k_r^2}\right)^{T_{2A}} \frac{(K_1^d)^{-\overline{T_1S}}(K_2^d)^{-\overline{T_2S}}}{\overline{T_1S}!\overline{T_2S}!(S_T - \overline{T_1S} - \overline{T_2S})!T_{1A}!(T_{1T} - T_{1A} - \overline{T_1S})!T_{2A}!(T_{2T} - T_{2A} - \overline{T_2S})!}$$

with $K_1^d = k_-^1/k_+^1$, $K_2^d = k_-^2/k_+^2$ and

(S16)
$$N = \sum_{\overline{T_1S}=0}^{min(T_{1T},S_T)} \sum_{\overline{T_2S}=0}^{min(T_{2T},S_T-\overline{T_1S})} \sum_{T_{1A}=0}^{T_{1T}-\overline{T_1S}} \sum_{T_{2A}=0}^{T_{2T}-\overline{T_2S}} \frac{\left(\frac{k_f^1 c_s}{k_r^1}\right)^{T_{1A}}\left(\frac{k_f^2 c_s}{k_r^2}\right)^{T_{2A}}(K_1^d)^{-\overline{T_1S}}(K_2^d)^{-\overline{T_2S}}}{\overline{T_1S}!\overline{T_2S}!(S_T - \overline{T_1S} - \overline{T_2S})!T_{1A}!(T_{1T} - T_{1A} - \overline{T_1S})!T_{2A}!(T_{2T} - T_{2A} - \overline{T_2S})!} \quad .$$

Finally, given the analytic solution of the master equation for the case with pure intrinsic noise, the full probability distribution in presence of extrinsic noise can be obtained in the usual way as a weighted superposition of conditional probabilities:

$$P(T_{1A}, T_{2A}, \overline{T_1 S}, \overline{T_2 S}) = \sum_{S_T=0}^{\infty} P(T_{1A}, T_{2A}, \overline{T_1 S}, \overline{T_2 S} | S_T) P(S_T) \quad . \tag{S17}$$

References

[1] T. M. Cover and J. A. Thomas, *Elements of information theory*. John Wiley & Sons. (2012).

[2] N. G. Van Kampen, *Stochastic processes in physics and chemistry*, vol. 1. Elsevier. (1992).
